# Missing Genes, Multiple ORFs, and C-to-U Type RNA Editing in *Acrasis kona* (Heterolobosea, Excavata) Mitochondrial DNA

**DOI:** 10.1093/gbe/evu180

**Published:** 2014-08-21

**Authors:** Cheng-Jie Fu, Sanea Sheikh, Wei Miao, Siv G.E. Andersson, Sandra L. Baldauf

**Affiliations:** ^1^Program in Systematic Biology, Department of Organismal Biology, Evolutionary Biology Centre, Uppsala University, Sweden; ^2^Key Laboratory of Aquatic Biodiversity and Conservation, Institute of Hydrobiology, Chinese Academy of Sciences, Wuhan, China; ^3^Department of Molecular Evolution, Cell and Molecular Biology, Science for Life Laboratory, Biomedical Centre, Uppsala University, Sweden

**Keywords:** Discoba, endosymbiotic gene transfer, horizontal gene transfer, codon usage bias, plant-type RNA editing, split ribosomal protein gene

## Abstract

Discoba (Excavata) is an ancient group of eukaryotes with great morphological and ecological diversity. Unlike the other major divisions of Discoba (Jakobida and Euglenozoa), little is known about the mitochondrial DNAs (mtDNAs) of Heterolobosea. We have assembled a complete mtDNA genome from the aggregating heterolobosean amoeba, *Acrasis kona*, which consists of a single circular highly AT-rich (83.3%) molecule of 51.5 kb. Unexpectedly, *A. kona* mtDNA is missing roughly 40% of the protein-coding genes and nearly half of the transfer RNAs found in the only other sequenced heterolobosean mtDNAs, those of *Naegleria* spp. Instead, over a quarter of *A. kona* mtDNA consists of novel open reading frames. Eleven of the 16 protein-coding genes missing from *A. kona* mtDNA were identified in its nuclear DNA and polyA RNA, and phylogenetic analyses indicate that at least 10 of these 11 putative nuclear-encoded mitochondrial (NcMt) proteins arose by direct transfer from the mitochondrion. *Acrasis kona* mtDNA also employs C-to-U type RNA editing, and 12 homologs of DYW-type pentatricopeptide repeat (PPR) proteins implicated in plant organellar RNA editing are found in *A. kona* nuclear DNA. A mapping of mitochondrial gene content onto a consensus phylogeny reveals a sporadic pattern of relative stasis and rampant gene loss in Discoba. Rampant loss occurred independently in the unique common lineage leading to Heterolobosea + Tsukubamonadida and later in the unique lineage leading to *Acrasis.* Meanwhile, mtDNA gene content appears to be remarkably stable in the *Acrasis* sister lineage leading to *Naegleria* and in their distant relatives Jakobida*.*

## Introduction

Among the three supergroups of eukaryotes, Excavata is by far the least well characterized (Adl et al. 2012; He et al. 2014). This includes the Discoba, the only excavates to possess respiratory competent mitochondria and mitochondrial DNA (mtDNA) (Simpson et al. 2006). Nonetheless, it is already apparent that the mtDNAs of the three to four major branches of Discoba—Jakobida, Euglenozoa, Heterolobosea, and probably Tsukubamonadida (Kamikawa et al. 2014)—exhibit a level of diversity unsurpassed by any other major eukaryotic lineage (Gray et al. 2004). For example, Jakobida have the most bacterial-like, gene-rich mtDNAs known, with 60–66 protein-coding genes with known functions (Burger et al. 2013). These include genes for a classical bacterial RNA polymerase, whereas all other known mtDNAs use a nuclear-encoded viral-type RNA polymerase (Lang et al. 1997). Meanwhile, euglenozoan mtDNAs are difficult to compare with those of other species due to extensive gene fragmentation (Flegontov et al. 2011), RNA editing (insertion/deletion) (Lukeš et al. 2005; Hajduk and Ochsenreiter 2010), and/or trans-splicing (Kiethega et al. 2011; Moreira et al. 2012).

Much less is known about the mtDNAs of Heterolobosea, which have only been characterized for two closely related species—*Naegleria gruberi* and *Naegleria fowleri* (Fritz-Laylin et al. 2011; Herman et al. 2013). These are among the most gene-rich mtDNAs outside of the jakobids, with 42 annotated and 4 hypothetical protein-coding genes (open reading frames [ORFs]). *Naegleria gruberi* is also the first organism outside of land plants found to encode DYW-type pentatricopeptide repeat (PPR) proteins (Knoop and Rüdinger 2010). These proteins are involved in organellar RNA editing (Lurin et al. 2004; Fujii and Small 2011; Yagi et al. 2013), and two sites of C-to-U RNA editing have been verified in the *N. gruberi* mitochondrion (Rüdinger et al. 2011). Lastly, the mtDNA of the only known tsukubamonad, *Tsukubamonas globosa*, has a gene content roughly similar to that of the two *Naegleria* species (Kamikawa et al. 2014).

Heterolobosea is a vast group consisting almost exclusively of unicellular amoebas or amoeboflagellates (Page and Blanton 1985; Harding et al. 2013). The only molecularly well-characterized taxon is *Naegleria* due to the medical importance of the opportunistic pathogen *N. fowleri*, which can cause fatal primary amoebic meningoencephalitis in humans (Visvesvara et al. 2007). The only completely sequenced heterolobosean is *N. gruberi*, a close relative of *N. fowleri* and a model organism for research on the microtubule cytoskeleton (Fritz-Laylin et al. 2010). Otherwise, there are no full genome sequences available for any other heterolobosean despite their abundance and ecological diversity (Cavalier-Smith and Nikolaev 2008; Yubuki and Leander 2008; Park and Simpson 2011; Pánek et al. 2012; Harding et al. 2013).

The Acrasidae are the only even quasi-multicellular heteroloboseans (Brown, Kolisko, et al. 2012). These are common soil microbes that spend most of their life cycle as free-living amebae. However, when food is depleted, the amebae can aggregate and cooperate to form small multicellular tree-like fruiting bodies (Brown, Silberman, et al. 2012). As this aggregative behavior resembles that of the dictyostelid slime molds (Amoebozoa), dictyostelids were originally classified together with acrasids as their sister taxon in the family Acrasidae (Olive 1970). However, Olive (1970) also noted striking differences in the morphology of their amoeboid stages. Molecular phylogenies now clearly assign the dictyostelids to the eukaryotic supergroup Amorphea, along with Metazoa and Fungi, and place acrasids on the opposite side of the tree in Heterolobosea (Adl et al. 2012; He et al. 2014).

We have assembled a complete *Acrasis kona* mtDNA genome using a combination of shot-gun and Sanger sequencing with long range polymerase chain reaction (PCR), along with a draft nuclear genome and transcriptome. The *A. kona* mtDNA has lost nearly 40% of the protein-coding genes identified in *Naegleria* mtDNA, most of which are found in *A. kona* nuclear DNA and polyA transcripts. In place of these missing genes, over one-fourth of the *A. kona* mtDNA consists of novel ORFs, while the remaining protein-coding sequences exhibit extensive reorganization, such as gene splitting and transposition and gene cluster reshuffling. C-to-U type editing of mitochondrial RNA is also identified in *A. kona*, along with the presence of DYW-type PPR proteins encoded in the nucleus. Mapping of gene presence/absence onto a consensus phylogeny reveals a sporadic pattern of gene loss and genome reorganization in Discoba*.*

## Materials and Methods

### Cell Culture and DNA Extraction

*Acrasis kona* ATCC strain MYA-3509 (formerly *Acrasis rosea*) (Brown, Silberman, et al. 2012) was grown on CM+ (Corn Meal Plus) agar, with streaked *Saccharomyces cerevisiae* as the food source. For DNA isolation, cells were grown in Spiegel’s liquid medium (Spiegel 1982) in 250-ml flasks and shaken on a rotary shaker (120 cycles/min) at room temperature. Cells were harvested in 50-ml corning tubes after 48 h at a cell density of approximately 1 × 10^5^/ml. Cell suspensions were transferred to Petri dishes and left for at least 1 h to allow the *A. kona* cells to settle and attach to the bottom. Cells were then washed three times with 10 mM phosphate buffer to remove the pellets of flocculated yeast. Cells were harvested by centrifugation and the DNA was extracted using the Blood & Cell Culture DNA Kit (Qiagen).

### mtDNA Sequencing

A large portion of the *A. kona* mtDNA sequence was recovered from 454 shot-gun sequencing of total *A. kona* DNA (Fu C-J, Sheikh S, Baldauf SL, unpublished data). Four contigs of mtDNA sequence with size ranges from 3 to 17 kb were obtained by genomic assembly using Newbler v2.5 (Roche). These contigs were used for a baiting and iterative mapping approach using Illumina sequencing data to correct base-calling errors known to be associated with long single-nucleotide repeats in 454 reads with Mira (Hahn et al. 2013). Long range PCR was carried out using nested primers to cross gap regions using the LongAmp Taq PCR Kit (NEB) (supplementary table S1, Supplementary Material online). PCR products ranging from 2 to 7 kb were cloned using the CloneJET PCR Cloning Kit (Fermentas) (supplementary fig. S1, Supplementary Material online). Colonies containing inserts were sent for sequencing with ABI 3730 sequencer (Applied Biosystems) at Macrogen (Seoul, South Korea). Final gap closure was accomplished using a primer walking strategy.

### RNA Extraction and Transcriptome Sequencing

RNA extraction and transcriptome sequencing was conducted as described in He et al. (2014). Briefly, cells were grown in Spiegel’s liquid medium and total RNA was extracted using TRI Reagent LS (Sigma-Aldrich). Poly (A)^+^ RNA was purified from 80 µg of total RNA using PolyAtract mRNA Isolation Systems (Promega) and sent for sequencing on a 454 GS FLX+ Titanium platform at Macrogen. Transcripts were assembled separately using the programs Newbler (v2.5, Roche) and Mira (v3.4) after removal of adapter sequences, and the results were combined using the program CAP3 (Huang and Madan 1999).

### Genome Annotation

ORFs were annotated using BLASTp and PSI-BLASTp searches of the National Center for Biotechnology Information (NCBI) nr database. For ORFs lacking significant hits (*E* value cutoff = 1e^−^^10^), the more sensitive HHpred method, which uses profile Hidden Markov Models (HMMs), was used to search against all the databases provided on its web server (http://toolkit.tuebingen.mpg.de/hhpred/, last accessed September 1, 2014) (Söding 2005). The validity of hits from bacteria or viruses was checked by positional conservation patterns based on multiple alignments from the Conserved Domain Database at NCBI where available. Structural RNAs and potential introns were predicted using the automated gene annotation tool MFannot (http://megasun.bch.umontreal.ca/cgi-bin/mfannot/mfannotInterface.pl, last accessed September 1, 2014) and the warnings in the output (e.g., alternative translation initiation sites, gene fusions, and exon–intron boundaries) were manually checked. Predicted boundaries of small and large ribosomal subunit RNA genes were verified in alignment with sequences from both *Naegleria* and jakobid mtDNAs. Transfer RNA (tRNA) genes were identified using tRNAscan-SE v1.23 (Lowe and Eddy 1997). Secondary structure of the largest noncoding mtDNA region was inferred with the MFold web server (Zuker 2003) using default settings and drawn with VARNA (Darty et al. 2009). Approximate tandem repeats were identified with tandem repeats finder (Benson 1999). The genome map was illustrated using DNAPlotter (Carver et al. 2009), followed by manual adjustment.

### Gene Synteny

The mtDNAs of discobid species listed in table 1 were used for reciprocal BLASTn and tBLASTx searches to identify regions of similarity, insertions, and rearrangements. Artemis (Rutherford et al. 2000) and Artemis Comparison Tool (ACT) (Carver et al. 2005) were used to interactively visualize the genomic regions of interest. A cutoff score of 40 was used to determine the presence/absence of gene synteny blocks. The gene order of the ribosomal protein (r-protein) synteny block between representative alpha proteobacteria and discobid species was identified by reciprocal BLASTp search with a threshold of 1e^−^^10^ and visualized with Circoletto (Darzentas 2010).

**Table 1:** General Features of *Acrasis kona* and Other Discoba mtDNAs

Species^a^	Size (bp)	AT Content (%)				AT Skew	GC Skew	Size Portion (%)		
		PCGs^b^	RNA^c^	Noncoding	Overall			PCGs	RNA	Noncoding
Heterolobosea										
*Acrasis kona*	51,458	84.0	72.4	89.8	83.3	0.102	0.354	82.5	10.7	6.8
*Naegleria gruberi*	49,843	79.3	67.1	85.0	77.8	−0.087	0.170	81.2	11.9	6.9
*Naegleria fowleri*	49,531	75.9	60.5	83.2	74.8	−0.079	0.196	79.9	11.6	8.5
Jakobida										
*Andalucia godoyi*	67,656	64.3	51.3	69.4	63.7	−0.012	0.015	81.0	10.2	8.8
*Histiona aroides*	70,177	64.7	52.5	67.2	64.6	−0.308	0.047	81.2	9.7	9.1
*Jakoba bahamiensis*	65,327	68.3	54.0	76.8	67.8	−0.102	0.138	82.1	10.1	7.8
*Jakoba libera*^d^	100,252	67.8	55.5	72.1	68.0	0.041	−0.017	72.0	7.0	21.0
*Reclinomonas americana*	69,586	73.7	55.3	83.2	73.2	−0.023	0.125	80.2	9.8	10.0
*Seculamonas ecuadoriensis*	69,158	68.2	53.9	76.8	68.1	0.017	−0.011	77.8	9.7	12.5
Tsukubamonadida										
*Tsukubamonas globosa*	48,463	67.4	56.5	70.6	66.2	−0.136	0.100	76.8	13.7	9.5

^a^GenBank accession number: *A. kona* KJ679272; *N. gruberi* AF288092; *N. fowleri* JX174181; *A. godoyi* KC353352; *H. aroides* KC353353; *J. bahamiensis* KC353354; *J. libera* KC353355; *R. americana* KC353356; *S. ecuadoriensis* KC353359; *T. globosa* AB854048.

^b^Putative coding regions including annotated protein-coding genes and unknown ORFs.

^c^Heterolobosea and Tsukubamonadida (rRNA and tRNA); Jakobida (rRNA, tRNA, RNase P-RNA, and tmRNA).

^d^mtDNA is linear.

### Codon Usage Analysis

For codon usage bias analysis, the values of expected effective number of codons (ENC or *Nc*) from GC content at synonymous third codon position (GC3s) under H0 (null hypothesis, i.e., no selection) were calculated according to the equation of Wright (1990): *Nc* = 2 + S + {29/[S^2 ^+ (1 − S)^2^]} (S denotes GC3s). If a given gene is only subject to G + C composition mutation constraint, it will lie just on the standard curve, whereas other kinds of selection and/or mutation pressure will cause values to lie above or below the curve (Fu et al. 2009). We also used its variant *Nc′*, which accounts for background nucleotide composition, to quantify bias in codon usage for individual genes in different mtDNAs using ENCprime (Novembre 2002). The measurement of codon deviation coefficient (CDC) was performed to investigate the potential influence of nucleotide positional heterogeneity on codon usage bias (Zhang et al. 2012), using the Composition Analysis Toolkit (CAT) with a statistical test of bootstrap resampling (10,000 replicates) under the default settings. GC content at first, second, and third codon positions (GC1, GC2, and GC3) was calculated for all annotated and unidentified ORFs with over 100 codons using codonW (codonw.sourceforge.net/) and visualized with GC Frame plot (watson.nih.go.jp/∼jun/cgi-bin/frameplot.pl). The two-sided Wilcoxon rank sum test was used to check the distribution of differences for the values of *Nc′* and GC3s between mtDNAs. All the statistical analyses were carried out in R (www.R-project.org, last accessed September 1, 2014).

### Amino Acid Compositional Homogeneity

For posterior predictive tests of compositional heterogeneity using PhyloBayes 3.2 (Lartillot et al. 2009), we used a concatenated protein data set consisting of 19 mtDNA-encoded proteins for 54 taxa (35 eukaryotes + 19 proteobacteria) including a total of 5,791 aligned amino acid positions (Burger et al. 2013). We tested the full data set and also recoded the amino acid data into the six Dayhoff groups (AGPST, C, DENQ, FWY, HKR, and ILMV) that tend to replace one another (Dayhoff et al. 1978). The tests use the default CAT model and run for 10,000 cycles, with first 5,000 cycles discarded as burn-in. The *z* score was used as a measure of the compositional deviation of individual taxa between the taxon-specific and global empirical frequencies over the 20 amino acids.

### Identification of Nuclear-Encoded Mitochondrial Protein Genes

Protein-coding genes uniquely missing from *A. kona* mtDNA relative to other discobids were searched for in the *A. kona* draft nuclear genome by tBLASTn using protein sequences from the *Naegleria* and jakobid mtDNA as queries. All hits with an *E* value < 1e^−10^ were examined by protein multiple sequence alignment with corresponding *Naegleria* and jakobid sequences. Assembly coverage plots of *A. kona* nuclear contigs were checked for the corresponding loci of all predicted nuclear-encoded mitochondrial (NcMt) gene regions using Tablet (Milne et al. 2013). Transcriptional activity of the putative NcMt genes was checked by direct mapping of the 454 transcriptome reads by Newbler (v2.5, Roche) and by BLASTn against the assembled mRNA transcript sequences. Mitochondrial transit peptide sequences and the N-terminal cleavage sites were predicted using TargetP (Emanuelsson et al. 2007), Predotar (Small et al. 2004), and Mitoprot (Claros and Vincens 1996).

### C-to-U RNA Editing Site Prediction and cDNA Synthesis

C-to-U type RNA editing sites in *A. kona* mtDNA were predicted using PREPACT (Lenz and Knoop 2013) (last accessed May 15, 2014). The probability of each candidate editing site was calculated by the percentage of the overlapping predictions against all the references from the output “commons.” Multiple alignment of the gene sequences containing the candidate sites was further checked. Oligonucleotide primer pairs were designed to flank the coding regions of four *A. kona* mitochondrial genes (*nad1*, *atp6*, *cob*, and *cox3*) with strong candidate sites (supplementary table S1, Supplementary Material online). For cDNA synthesis, total RNA was extracted and treated with DNase (Thermo). First strand cDNA was synthesized using the Phusion RT-PCR Kit (Thermo) with hexanucleotide random primer mix. PCR amplification of both mitochondrial genomic sequence and cDNA products was performed using the Phusion High-Fidelity DNA Polymerase (Thermo). PCR amplicons were cleaned with ExoSap-IT (GE Healthcare) and sent for direct sequencing.

### Identification of DYW-Type PPR Proteins

Known DYW-type PPR protein sequences in *N. gruberi* (11) and *Physcomitrella patens* (10) (Knoop and Rüdinger 2010) were used as queries to search against the *A. kona* nuclear contigs, either with full length protein sequences (including variable length PLS repeat domains) or using only the conserved carboxy-terminal E/E+/DYW domain as query. All candidate proteins found were screened for the possible presence of PPR motifs using TPRpred (Karpenahalli et al. 2007). Homologous sequences were obtained by taxon-limited BLASTp searches of the NCBI database against all major groups of early branching land plants (Liverworts, Mosses, Hornworts, and Lycophytes) and additional taxa based on (Iyer et al. 2011; Schallenberg-Rüdinger et al. 2013), specifically Heterolobosea (*N. gruberi*), Amoebozoa (*Acanthamoeba castellanii*, *Physarum polycephalum*), Metazoa (*Adineta riccia*, *Philodina roseola*), Fungi (*Laccaria bicolor*), Charophyta (*Nitella hyaline*), and *Malawimonas jakobiformis*. Only sequences containing full E/E+/DYW domains and/or with conservative key amino acid positions were used in subsequent analyses. Sequence logos were generated using WEBLOGO (Crooks et al. 2004).

### Phylogenetic Analyses

For putative *A. kona* NcMt genes, single gene trees were generated from inferred amino acid sequences aligned with MAFFT v7 (Katoh and Standley 2013). For the phylogeny of discobids, a concatenated data set of 24 mitochondrial proteins (Atp1, 3, 6, 8, 9, Cox1, 2, 3, 11, Cob, Nad1, 2, 3, 4, 4L, 5, 6, 7, 8, 9, 10, 11, Sdh2, and TufA) was used from taxa with complete mtDNA sequences. Conserved blocks were extracted using Gblocks with relaxed parameters (Castresana 2000). Multiple sequence alignment files are available upon request. Bayesian analysis was performed on NcMt proteins with MrBayes v3.2.2 (Ronquist et al. 2012) using a mixture of amino acid models. Searches consisted of two sets of four chains run over 1 million generations, discarding a burn-in of 25%. Bayesian analysis of discobid phylogeny and DYW-type PPR protein was performed with PhyloBayes MPI 1.4f (Lartillot et al. 2013), using the CAT + Gamma model and the predefined WLSR5 profile model (Wang et al. 2008), respectively, with constant sites removed (-dc). Analyses were run for at least 15,000 cycles (Max diff < 0.20), with the first 5,000 cycles discarded as burn-in. Maximum-likelihood analysis was conducted with RAxML v7.3.3 (Stamatakis et al. 2012) using the PROTGAMMALG model and the fast bootstrapping option (1,000 replicates). All phylogenetic analyses were run on the CIPRES Science Gateway (www.phylo.org, last accessed September 1, 2014).

## Results

### General Features, Gene Content, and Genome Organization in *A. kona* mtDNA

**Fig. 1.—:**
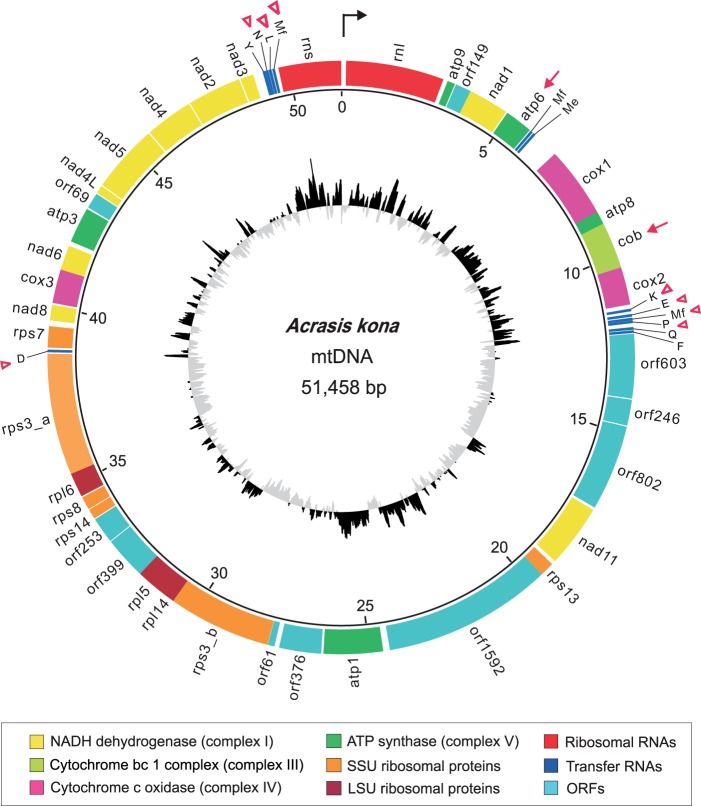
The mitochondrial genome of *Acrasis kona*. A circular map of the *A. kona* mtDNA is shown, drawn to scale as indicated by the inner circle and with coordinates in kilobases. The outermost track shows predicted genes, color-coded by functional category as indicated in the box below the map. The innermost circle shows GC content above (black) or below (gray) the genome average. Protein-coding genes with identified C-to-U type RNA editing are indicated with arrows. tRNA genes with predicted mismatches at the first three positions of the acceptor stem are indicated with triangles.

The *A. kona* mitochondrial genome was assembled into a single circular-mapping molecule with a size of 51,458 bp (fig. 1). Its overall A + T (AT) content is 83.3%, higher than those of the two *Naegleria* mtDNAs (74.8–77.8%), the only other sequenced heterolobosean mtDNAs. In fact, *A. kona* mtDNA has the highest AT content among known mtDNAs of free-living discobids for noncoding regions (89.8% AT), protein-coding genes (84% AT), and structural RNA genes (72.4% AT) (table 1). Despite this extreme AT-richness, *A. kona* mtDNA is predicted to consist of 93.2% coding regions, placing it among the most compact discobid mtDNAs, along with *N. gruberi* (93.1%) and *Jakoba bahamiensis* (92.2%), the most compact jakobid mtDNA (table 1). The largest noncoding region (755 bp) of *A. kona* mtDNA also contains its largest repetitive region, which exhibits extensive predicted secondary structure (supplementary fig. S2, Supplementary Material online).

**Fig. 2.—:**
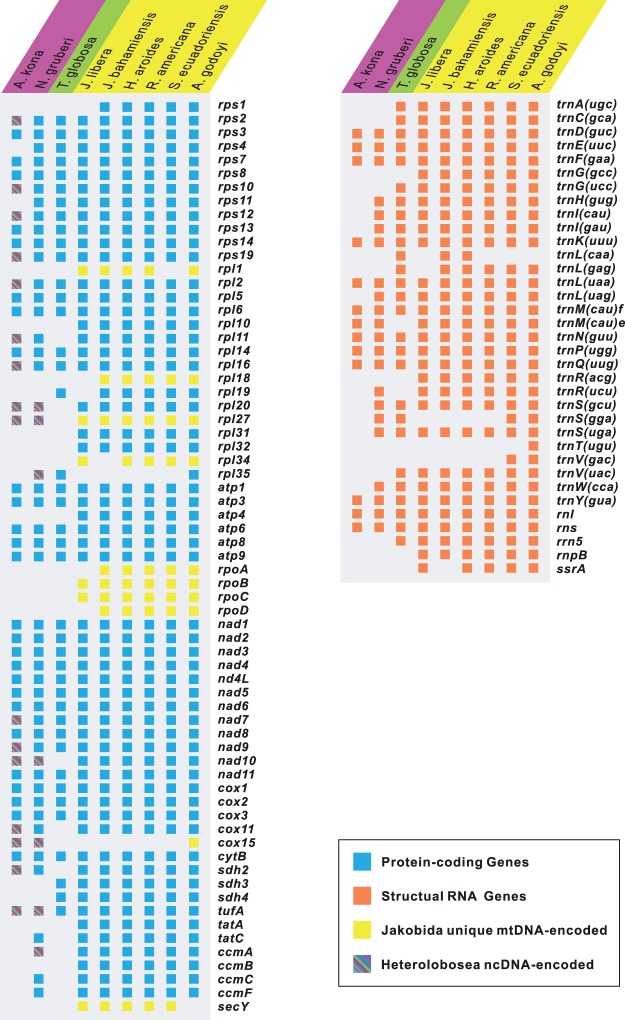
A comparison of mtDNA gene contents among representative lineages of Discoba. Taxa names are shaded in purple (Heterolobosea), green (Tsukubamonadida), and yellow (Jakobida). Genes are color-coded according to the key in the box at the bottom right. mtDNA-encoded proteins exclusively found in Jakobida are indicated according to Burger et al. (2013). Nuclear-encoded mitochondrial (NcMt) proteins of two heterolobosean species (*Acrasis kona* and *Naegleria gruberi*) were identified using jakobid mtDNA genes as query (tBLASTn, *E* value < 1 e^−10^).

Despite its high predicted coding capacity, the number of protein-coding sequences with known functions in *A. kona* mtDNA is markedly less than in *Naegleria*, as are the number of predicted tRNA genes (fig. 2). Only 26 protein-coding genes and 11 different tRNA genes can be identified in *A. kona* mtDNA, versus 42 protein-coding genes and 20 tRNAs in both examined *Naegleria* mtDNAs and 41 protein-coding genes and 24 tRNAs in *Tsukubamonas* (fig. 2). We compared the sequence divergence of oxidative phosphorylation (OXPHOS) pathway genes in representative discobid mtDNAs (supplementary table S2, Supplementary Material online), as these are nearly universal among functional mitochondria and generally well conserved (Szklarczyk and Huynen 2010). The two *Naegleria* species have an overall divergence of 18.2% for these proteins at the amino acid level, which is similar to that between the two most closely related jakobids, *Histiona aroides* and *Reclinomonas americana* (16.2%). For comparison, there is 59.0% overall divergence between OXPHOS genes shared by *Acrasis* and *Naegleria*, reflecting their more distant relationship.

**Fig. 3.—:**
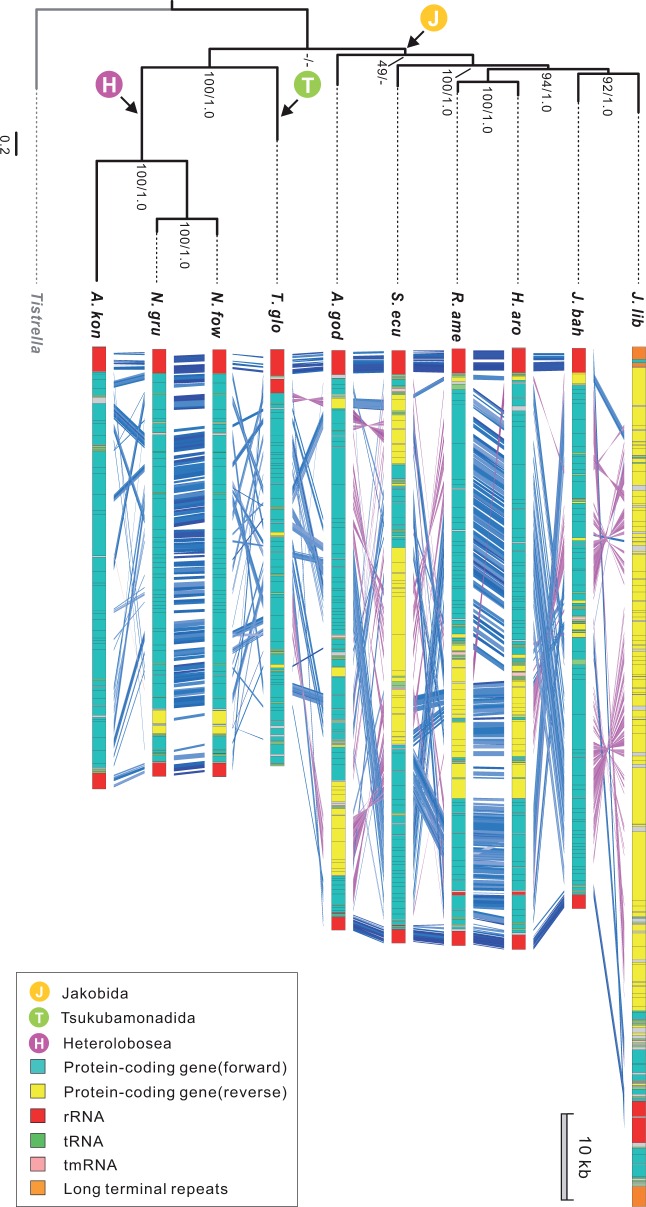
A mapping of overall mtDNA gene synteny between different lineages of Discoba (Heterolobosea, Tsukubamonadida, and Jakobida) onto a consensus phylogeny. The tree was based on a concatenated data set of 24 mitochondrial proteins (7,313 aligned amino acid positions), with the α-proteobacterium *Tistrella mobilis* (NC_017956) as outgroup. Numbers indicate maximum-likelihood bootstrap values with RAxML (Stamatakis et al. 2012) and posterior probabilities with PhyloBayes (Lartillot et al. 2013). Genes are color-coded by functional category as indicated in the box. Genes in the forward versus reverse order are indicated in blue and purple lines, respectively, as generated by ACT (Carver et al. 2005) with a cut off value of 40.

A mapping of the overall genome synteny among discobid species onto a consensus phylogeny of the taxa shows that the mtDNAs of the two closely related *Naegleria* species have a highly conserved gene order. In contrast, very little synteny is observed between *Acrasis* and *Naegleria* mtDNA (fig. 3). Good overall conservation of synteny is also seen between terminal clades of jakobids, although considerable gene transposition and inversion are found between the mtDNAs of more distantly related jakobids, especially between the earliest branches of the clade (fig. 3). One exception to this is four genes encoding NADH dehydrogenase subunits (*nad4L*, *nad5*, *nad4*, and *nad2*) (fig. 1). These share the same gene order in *Acrasis*, *Tsukubamonas* and all six jakobids, although they are dispersed in the *Naegleria* mtDNAs*.*

### A Highly Interrupted Ribosomal Protein Gene Cluster

Of special interest is a highly conserved gene cluster recently identified in all jakobid mtDNAs, which corresponds to a large synteny block of r-protein operons (Burger et al. 2013). The order of these in the free-living α-proteobacterium *Tistrella mobilis* is (L11–L10–Beta–Str–S10–Spc–Alpha) (supplementary fig. S3, Supplementary Material online), which is thought to represent the ancestral organization of these operons in bacterial genomes (Yang and Sze 2008; Bratlie et al. 2010). We compared the arrangement of r-protein genes in heterolobosean and jakobid mtDNAs with those of close α-proteobacterial relatives, focusing particularly on *Rickettsia* where the L11–L10–Beta operons are separated from the Str–S10–Spc–Alpha operons but gene content and orders are well conserved within operons [supplementary fig. S3, Supplementary Material online; unlike (Burger et al. 2013)].

**Fig. 4.—:**
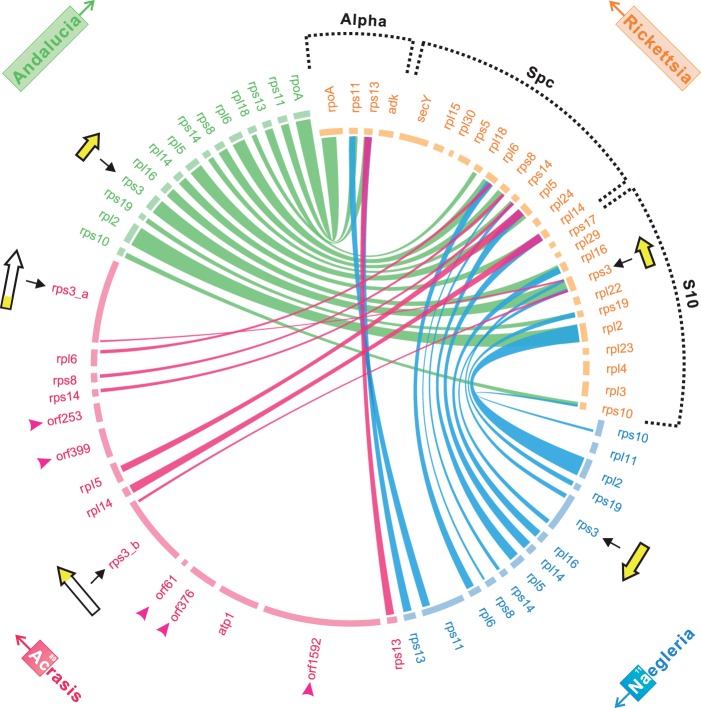
A comparison of mtDNA gene order for the largest r-protein synteny block. The same block is compared with the generally contiguous S10, spectinomycin (Spc) and Alpha operons of α-proteobacteria as determined by BLASTp (*E* value < 1 e^−10^) against *Rickettsia prowazekii* genome (NC_000963). The locations of the *rps3* genes within each synteny cluster are indicated with schematic models. The unknown ORFs in *Acrasis kona* are indicated with fuchsia triangles.

Gene order in the three contiguous r-protein operons—S10, Spc, and Alpha—is well conserved in *Naegleria* compared with *Tsukubamonas*, all jakobids, and α-proteobacteria (fig. 4). However, there are many fewer r-protein genes in *A. kona* mtDNA compared with *Naegleria* (8 vs. 17, respectively), and the only detectable gene synteny here in *A. kona* is in the Spc operon. The *A. kona* cluster further appears to be disrupted by the insertion of several putative protein-coding sequences in this synteny block, including *atp1* and multiple ORFs of unknown function (fig. 4).

Interestingly, in *A. kona* mtDNA the five genes identified in the Spc operon are flanked on both sides by genes showing similarity to the *rps3* gene, which is normally located inside the S10 operon (fig. 4). In *A. kona*, the gene is not only split, but the two halves designated as *rps3_a* and *rps3_b* are transposed relative to each other, separated by 4,588 bp that contains the five r-protein genes in the Spc operon plus two ORFs, and both reading frames are substantially extended (supplementary fig. S4, Supplementary Material online). Notably, the split occurs at roughly the same position as an approximately 300 aa insertion in *Naegleria* rps3. Together the two *A. kona* fragments encode a protein (2,099 aa) much larger than other known rps3 proteins (200–250 aa), and only the first 86 aa of rps3_a (1,133 aa) and the final 93 aa of rps3_b (966 aa) map to the predicted *Naegleria* rps3, at the N- and C-termini, respectively. GC content is also heterogeneous across the border between the conserved and extension portion of both fragments (supplementary fig. S4, Supplementary Material online). Moreover, the two genes flanking *rps3* in the jakobid and *Naegleria* mtDNAs, *rpl16* and *rps19*, both appear to be transferred to the nucleus in *A. kona* (see discussion).

### Acquisition of Novel ORFs

Despite its lower gene content, the *A. kona* mtDNA genome (51.5 kb) is roughly the same size as those of *Naegleria* (49.5–49.8 kb) and *Tsukubamonas* (48.6 kb). This is due to the presence of ten predicted ORFs in *A. kona* mtDNA, constituting 26.5% of the genome and potentially encoding proteins ranging in size from 69 to 1,592 amino acids (supplementary table S3, Supplementary Material online). All ten ORFs occur on the sense DNA strand and in the same transcriptional orientation as the rest of the coding content of the genome (fig. 1). No significant sequence similarity (tBLASTx, *E* value threshold 1 e^−^^10^) was detected between the *A. kona* ORFs and the four ORFs in *Naegleria* mtDNA or any of the ORFs found in other discobid mtDNAs. The HMM search of *A. kona* ORFs for domain similarities against multiple databases (Pfam, SCOP, and InterPro) also did not generate significant hits (*E* value threshold 1 e^−^^3^).

**Fig. 5.—:**
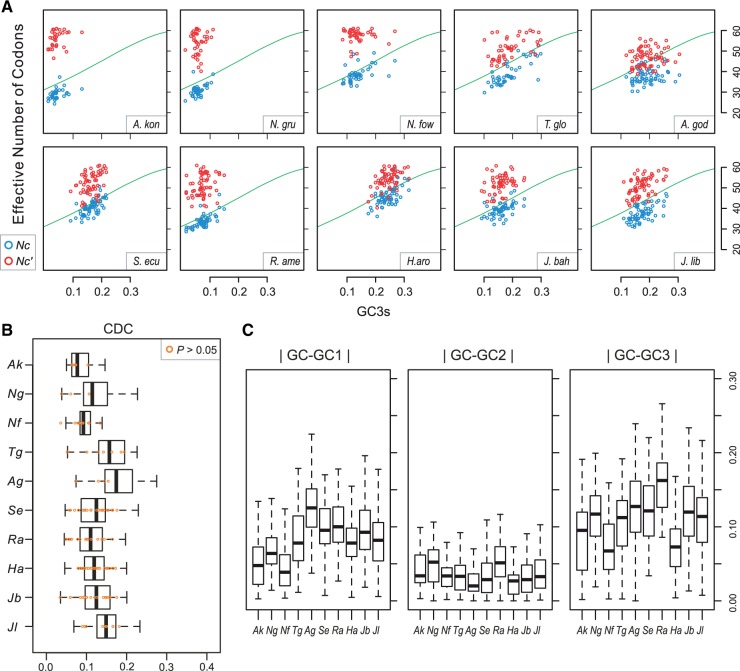
Codon usage bias and nucleotide positional heterogeneity of mitochondrial protein-coding sequences in the mtDNAs of Discoba. (*A*) The values of *Nc* (Wright 1990) and its variant (*Nc′*) (Novembre 2002) are plotted against GC3s. A line of standard curve based on the equation of Wright (1990) is superimposed on the graph. (*B*) Values of codon deviation coefficient (CDC) (Zhang et al. 2012) range from 0 (no bias) to 1(maximum bias). Genes with a CDC value of nonstatistical significance (*P* > 0.05) are indicated in orange circles. (*C*) Heterogeneity of positional GC content is represented by absolute differences between overall GC content and positional GC content at first, second, and third codon positions (GC1, GC2, and GC3). Ak, *Acrasis kona*; Ng, *Naegleria gruberi*; Nf, *Naegleria fowleri*; Tg, *Tsukubamonas globosa*; Ag, *Andalucia godoyi*; Ha, *Histiona aroides*; Jb, *Jakoba bahamiensis*; Jl, *Jakoba libera*; Ra, *Reclinomonas americana*; Se, *Seculamonas ecuadoriensis*.

Codon usage bias analysis of the novel ORFs could show whether the putative genes have been exposed to the same mutation pressure as the more typical mitochondrial genes (e.g., OXPHOS pathway), and thus provide an indication of when these novel genes were acquired. A plot of *Nc* versus GC3s shows that values for all *A. kona* genes have generally small distance deviations from the standard curve (fig. 5*A*). When corrected for background nucleotide composition, the *Nc′* values for most *A. kona* genes range from 50 to 61(overall 54.10) (fig. 5*A*). The calculated overall value of CDC of *A. kona* genes (0.091 ± 0.029) was also shown to be the lowest among all discobid mtDNAs (fig. 5*B*). Thus, the measurement of *Nc*, *Nc*′, and CDC all suggest no strong observed codon usage bias throughout *A. kona* mtDNA, likely reflecting a weak selection pressure on this genome despite its overall high AT content. In fact, variation in the heterogeneities of positional GC contents, particularly at first and third codon positions appears to be generally consistent with overall estimated codon usage bias in protein-coding sequences throughout discobid mtDNAs (fig. 5*C*).

The codon position-specific GC content of the *A. kona* ORFs is generally consistent with the rest of the protein-coding genes in its mtDNA (supplementary table S3, Supplementary Material online), and no significant difference was detected for *Nc′* or GC3s between the protein-coding sequences and the unknown ORFs in *A. kona* (Wilcoxon rank sum test, *P* = 0.483 and 0.263, respectively). This suggests that these ORFs have resided in the *A. kona* mtDNA for a long enough time to adjust the base composition pattern to the strong AT mutation pressure. However, the largest ORF (*ORF1592*) has the highest GC content value at third codon position of all mitochondrial genes (0.163), which is about 2- to 3-fold higher than most other genes. That is largely due to a number of repeat elements in *ORF1592*, including two tandem repeats with a periodicity of 9 and 18 bp and heterogeneous GC3 content (supplementary fig. S2, Supplementary Material online). Meanwhile, *ORF603*, *ORF246*, and *ORF802* tend to be lower in GC at both first and second codon positions than the typical mitochondrial genes, perhaps indicating that they evolve under lower selective constraints than the other genes (supplementary table S3, Supplementary Material online).

To examine whether lowered selective constraints have resulted in a more extreme amino acid bias in *A. kona* mtDNA, we examined the amino acid compositional heterogeneity for a set of 19 mitochondrial proteins (mainly involved in OXPHOS pathway) among a broad sampling of taxa (54 species). The compositional deviation for *A. kona* is among the highest detected of all taxa (supplementary table S4, Supplementary Material online), indicating a substantial violation of homogeneity (Lartillot et al. 2009). When these protein sequences were recoded using the six Dayhoff common amino acid substitution groups (Dayhoff et al. 1978), the *z* scores are decreased for most examined individual taxa except for *Acrasis* or *Naegleria* or for a scattered sampling of other taxa, particularly some amoebozoan species (*Acantamoeba*, *Dictyostelium*, and *Hartmannella*) (supplementary table S4, Supplementary Material online). The presence of biased amino acid composition in the OXPHOS proteins of *A. kona* mtDNA could possibly be linked to several attributes mentioned above, that is, a combined outcome of extreme AT content, high coding sequence divergence, and a loose constraint of selection pressure.

### Functional Gene Transfer to the Nucleus

Among the 16 annotated mitochondrial protein-coding genes that are missing from *A. kona* mtDNA relative to *Naegleria*, 11 were detected as full predicted ORFs on *A. kona* nuclear contigs. The corresponding loci of each NcMt gene candidate on *A. kona* nuclear contigs were checked by coverage plots. Consistent homogeneous coverage patterns were shown for all these predicted genes, along with their flanking regions (supplementary fig. S5, Supplementary Material online). These 11 putative NcMt proteins include seven r-proteins, three OXPHOS proteins (*nad7*, *nad9*, and *sdh2*), and one protein involved in protein maturation (*cox11*) (supplementary table S5, Supplementary Material online). The missing genes in *A. kona* mtDNA include four from the S10 operon (*rps10*, *rps19*, *rpl2*, and *rpl16*), all of which are identified in *A. kona* nuclear contigs.

Phylogenetic trees for 10 of the 11 *A. kona* putative NcMt genes show them to be most closely related to their *Naegleria* mtDNA homologs (supplementary fig. S6, Supplementary Material online). This is despite the short lengths of many of these proteins (<130 aligned amino acid positions), which generally makes it difficult to get a strong phylogenetic signal. All of the 11 *A. kona* NcMt genes except *rps2* are strongly predicted to carry a mitochondrial targeting signal. Moreover, 9 of the 11 *A. kona* NcMt genes are also predicted to encode N-terminal extensions relative to their counterparts in *Naegleria* mtDNA, potentially indicating nucleus-derived mitochondrial transit peptides ranging in size from 7 to 61 amino acids (supplementary table S5, Supplementary Material online). However, no sequence similarity was detected among these predicted transit peptides at the nucleotide or amino acid level. Transcriptome data also show that all the 11 *A. kona* putative NcMt genes are actively transcribed into polyA RNA (data not shown). Thus it appears that these *A. kona* putative NcMt genes represent functional gene transfer from mtDNA to the nucleus, sometime since the last common ancestor (LCA) of *Acrasis* and *Naegleria*.

### C-to-U Type RNA Editing

**Fig. 6.—:**
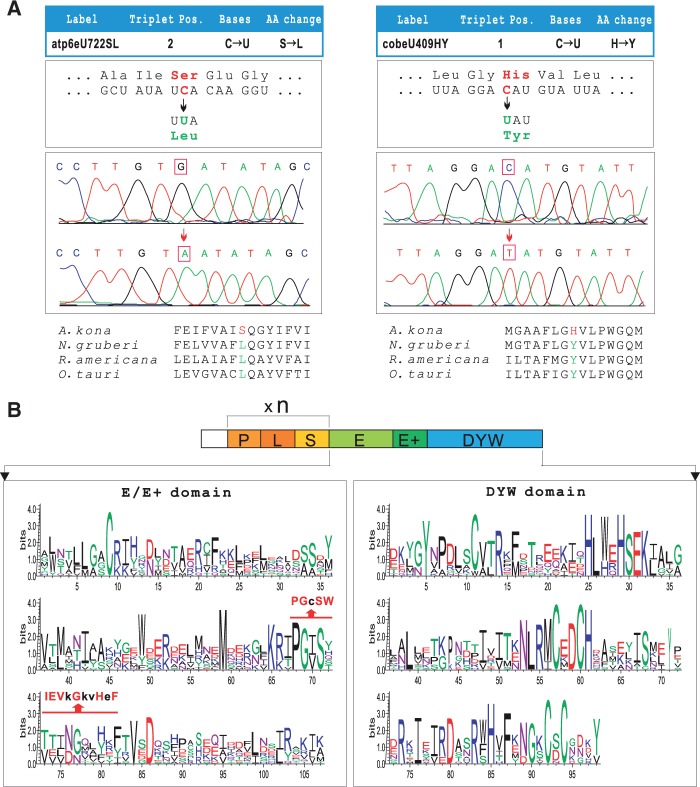
C-to-U mitochondrial RNA editing and the DYW-type PPR proteins in the *Acrasis kona* genome. (*A*) Top: Two RNA editing sites in *Acrasis* mtDNA-encoded genes (atp6eU722SL and cobeU409HY). Middle: The PCR and RT-PCR products from genomic and cDNA templates. The *Atp6* gene was sequenced using the antisense primer. Bottom: Conserved amino acid codons illustrated by alignments including *Naegleria gruberi* (Heterolobosea), *Reclinomonas americana* (Jakobida), and *Ostreococcus tauri* (green alga). (*B*) The schematic motif structure and plot of sequence conservation for the *Acrasis* DYW-type PPR proteins. A highly conserved 15 amino acid motif (PG box) identified in the land plants is indicated above the positions.

Sequence comparisons suggest the presence of six strong candidate sites of C-to-U RNA editing in four *A. kona* mtDNA genes—*nad1*, *atp6*, *cob*, and *cox3*. Editing was checked for all six sites by sequencing PCR products of the four genes from both genomic and cDNA templates. These experiments confirmed two of the predicted C-to-U editing sites, one each in the *atp6* and *cob* genes (atp6eU722SL and cobeU409HY) (fig. 6*A*), but rejected the remaining four.

We searched the *A. kona* nuclear contigs for potential homologs of the DYW-type PPR proteins, which have been postulated as the key factors in C-to-U RNA editing in plant organelles (Salone et al. 2007). This identified 12 predicted ORFs with a recognizable E/E+/DYW domain at their carboxy-terminus. These ORFs were all further predicted to encode multiple PLS repeat domains by the program TPRpred (Karpenahalli et al. 2007). A highly conserved 15 amino acid motif or “PG box” (PGxSWIEVxGxxHxF) that bridges the E and E+ domain, previously identified in land plant DYW-type PPR proteins (Okuda et al. 2007) is also present in the 12 *A. kona* ORFs (fig. 6*B*).

**Fig. 7.—:**
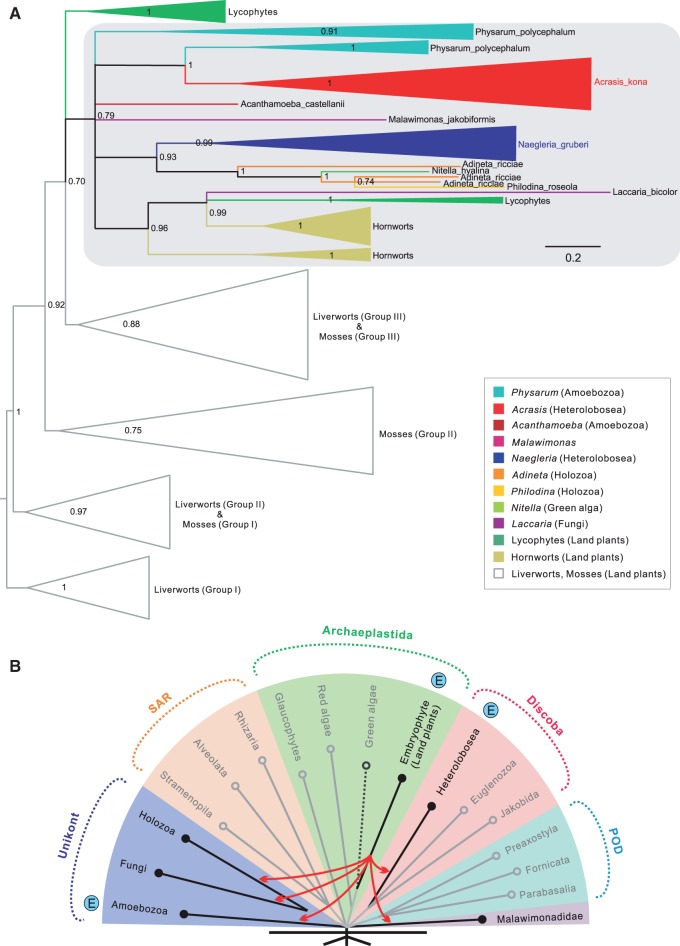
Phylogeny of DYW-type PPR proteins in the eukaryote species. (*A*) Bayesian phylogeny of the E/E+/DYW domain of the DYW-type PPR proteins. Taxa include a broad sampling of early branching land plants (Liverworts, Mosses, Hornworts, and Lycophytes), and those outside land plants (Heterolobosea [*Acrasis kona*, *Naegleria gruberi*], Amoebozoa [*Acanthamoeba castellanii*, *Physarum polycephalum*], Metazoa [*Adineta ricciae*, *Philodina roseola*], Fungi [*Laccaria bicolor*], Charophyta [*Nitella hyaline*], *Malawimonas jakobiformis*). Values correspond to posterior probabilities; all nodes with posterior probability less than 0.7 are collapsed. Taxon labels are color-coded according to the key at the bottom right. The strong phylogenetic affinity of the only known green algal sequence (*N. hyaline*) for rotifers may indicate possibly transcriptomic data contamination as reported in (Laurin-Lemay et al. 2012). A detailed list of taxa is shown in supplementary figure S7, Supplementary Material online. (*B*) A schematic tree showing the phylogenetic distribution of DYW-type PPR proteins in the major eukaryote lineages. Lineages with experimental evidence of C-to-U type RNA editing are indicated with “E.” Arrows indicate the probable HGT directions from land plants to the other eukaryotes. Dotted line indicates the doubtful presence of DYW-type PPR proteins in *N. hyaline*.

DYW-type PPR proteins have been identified in almost every major group of land plants except for marchantiid liverworts (Rüdinger et al. 2008), as well as in additional scattered taxa across the eukaryote tree of life (Knoop and Rüdinger 2010; Iyer et al. 2011; Schallenberg-Rüdinger et al. 2013). Phylogeny of the DYW-type PPR proteins further suggests that the genes encoding these proteins most probably have spread among eukaryotes through horizontal gene transfer (HGT) from early branching land plants (fig. 7*A* and *B*). Where found these sequences show a general trend toward lineage-specific expansion and diversification, including within *Acrasis* and *Naegleria* (supplementary fig. S7, Supplementary Material online). Interestingly, *Acrasis* DYW-type PPR proteins show a strong phylogenetic affinity for the distant-related amoebozoan *Physarum* (1.0 Bayesian posterior probability, biPP), whereas the homologues of *Naegleria* group together with the rotifer clade (0.93 biPP) (fig. 7*A*). This suggests possible multiple independent acquisition of the plant-type RNA editing factors in these two heterolobosean lineages.

## Discussion

### Gene Loss and Genome Rearrangement in *A. kona* mtDNA

A complete sequence of the *A. kona* mtDNA shows that it is roughly the same size as the mtDNA of its closest sequenced relatives (*Naegleria* spp.), but has a remarkably different organization and gene content (figs. 2 and 3). Sixteen genes of known function, corresponding to nearly 40% of protein-coding genes in *Naegleria* are missing from *A. kona* mtDNA. Eleven of these missing genes are found on *A. kona* nuclear contigs, and phylogenetic analyses suggest that at least 10 of these 11 putative NcMt genes arose by direct functional transfer of mtDNA to the nucleus (Timmis et al. 2004). Seven of the 11 putative transfers involve r-proteins, consistent with evidence that r-protein genes tend to be lost from mtDNA more often than respiratory genes (Adams and Palmer 2003). It thus would be of interest to look for the preinsertion nuclear loci where such functional transfer has occurred upon the completion of the annotation of *A. kona* draft nuclear genome. The translation products of five remaining missing genes (*rps4*, *rps11*, *ccmF*, *ccmC*, and *tatC*) in *A. kona* mtDNA are usually present in the mitochondrial proteomes of free-living species (Gray et al. 2004). Thus there may have been additional gene transfers in *Acrasis* that may be difficult todetect due to the small size and/or low sequence conservation of the missing genes, possibly further complicated by fragmentation due to intron insertion within the nuclear genome.

The general pattern of genome degradation in *A. kona* mtDNA also includes loss of nearly half (9 of 20) of its tRNA genes relative to *Naegleria* mtDNA. This implies the need for extensive import of tRNAs from the cytosol (Salinas et al. 2008; Lithgow and Schneider 2010). In addition, mismatches were identified in the first 1–3 bp of the amino acid acceptor stem in 8 of the 11 remaining *Acrasis* tRNAs (fig. 1), as well as in 8 of the 20 predicted tRNAs in *Naegleria* mtDNA (data not shown). Such tRNAs would require editing in order to create the standard Watson–Crick base pairing necessary for functional mature tRNAs, thus a second RNA editing system (e.g., mitochondrial 5′-tRNA editing; Jackman et al. 2012) would probably be required in addition to the mRNA editing system (see above). The *Acrasis* tRNAs are otherwise well conserved in sequences, suggesting that they are not simply pseudogenes functionally replaced by additional tRNA imported from the cytosol.

Transfer to the nucleus of at least nine r-proteins in *A. kona* also coincides with massive rearrangement of its r-protein operons relative to the bacterial-like organization of these operons in *Naegleria*, *Tsukubamonas,* and all six examined jakobids (fig. 4). Gene order is further disrupted by the insertion of novel ORFs, as well as splitting and extension of the *A. kona rps3* gene*.* Notably, *rps3* is flanked in jakobid and *Naegleria* mtDNAs by two genes that are transferred to the nucleus in *A. kona* (*rpl16* and *rps19*), allowing for the possibility that these phenomena may be linked. Splitting of the *rps3* genes has previously been documented in a number of eukaryotes, often accompanied by insertion of additional functional domains or long peptides (Swart et al. 2012).

### Mode and Tempo of Genome Evolution in Discoba

**Fig. 8.—:**
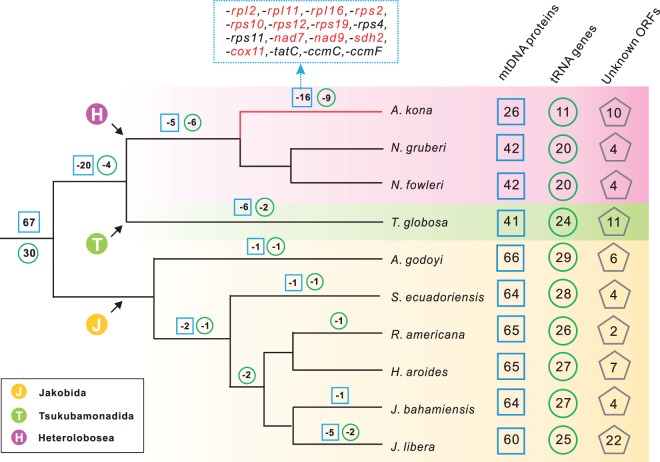
A mapping of mtDNA gene content onto a schematic phylogeny of Discoba, excluding Euglenozoa. Evolutionary loss of mtDNA gene content is indicated assuming the most parsimonious steps. Genes colored in red were identified on the *Acrasis kona* nuclear contigs (tBLASTn, *E* value < 1e^−10^).

A mapping of mtDNA gene content onto a consensus phylogeny reveals a sporadic pattern of relative stasis and rampant gene loss in Discoba (fig. 8). Altogether 67 protein-coding genes are widely distributed in jakobid mtDNA (Burger et al. 2013) and therefore presumably present in the LCA of Discoba. Assuming that these mitochondrial genes were directly inherited from the mtDNA of the eukaryote LCA and that *Tsukubamonas* is more closely related to Heterolobosea than to Jakobida (Kamikawa et al. 2014), this implies a loss of 20 protein-coding genes between the LCA of Discoba and the LCA of Heterolobosea + Tsukubamonadida (fig. 8). Following the latter split, the *Tsukubamonas* lineage lost only six additional protein-coding genes, similar to the ancestral lineage of *Acrasis* + *Naegleria*, which lost five protein-coding genes. However, within the clade of Heterolobosea, gene loss appears to have halted entirely in the lineage leading to *Naegleria*, whereas the lineage leading to *Acrasis* appears to have continued the process of endosymbiotic gene transfer at the same or even an accelerated pace (fig. 8). Thus a sporadic pattern of evolutionary stasis and accelerated mtDNA gene loss is observed in Heterolobosea, a phenomenon rare for free-living organisms within other major groups of eukaryotes (Lang et al. 1999; Adams and Palmer 2003), in this case beginning with a much more gene-rich mtDNA ancestor.

The Discoba also show a preponderance of unknown ORFs, ranging from 2 to 22, most of which are not shared by any two mtDNAs. Thus, gain and loss of ORFs appears to be a very dynamic process in Discoba (fig. 8)*.* However, these ORFs still constitute a small fraction of the potential protein-coding capacity of these mtDNAs, with the exception of *A. kona*, where ORFs constitute over a quarter (26.5%) of the genome, most of which are predicted to encode proteins over 200 amino acids in size. Several things suggest that the novel ORFs in *A. kona* mtDNA are probably functional genes. The ORFs exhibit an overall similar pattern of codon usage as the protein-coding sequences in the *A. kona* mtDNA, indicating that whatever their origin is, these ORFs have resided in the *A. kona* mtDNA for quite some time and have homogeneous base composition patterns as the rest of genome. Some of the ORFs also show faster evolution at third codon positions (supplementary table S3, Supplementary Material online). Transcripts corresponding to at least one ORF (*ORF1592*) were obtained by RT-PCR (data not shown). Investigating the potential significance of these ORFs in *Acrasis* will require sequencing and functional analysis of additional mtDNAs from acrasids and their close relatives.

### C-to-U Type of RNA Editing in Heterolobosea mtDNAs through Ancient HGT

Different RNA editing systems are found across eukaryotes, particularly in organelles (Knoop 2011; Gray 2012). DYW-type PPR proteins are postulated as the key specificity determinants in C-to-U type editing (Salone et al. 2007; Zehrmann et al. 2009; Hayes et al. 2013), and co-occurrence of the DYW domain and organelle RNA editing is well documented in land plants (Rüdinger et al. 2012). However, the 12 putative DYW-type PPR proteins found in the *A. kona* nuclear genome far exceed the two confirmed C-to-U editing sites in its mtDNA, although additional editing sites may exist among the unknown ORFs that are not readily predicted. Likewise, only two C-to-U RNA editing sites were identified by extensive transcriptome analysis in *N. gruberi* (Rüdinger et al. 2011) despite the presence of 11 putative DYW-type PPR proteins in its nuclear genome (Knoop and Rüdinger 2010). Low RNA-editing activity in these mtDNAs could possibly have resulted from their extremely low GC content (table 1) (Jobson and Qiu 2008). Thus the excess of DYW-type PPR proteins suggests that they may play other roles, such as organellar endonucleolytic cleavage (Okuda et al. 2009) or transcript splicing (Ichinose et al. 2012) or have cytoplasmic activities. The genes targeted for C-to-U RNA editing differ between *A. kona* and *N. gruberi*, indicating that target sites are not highly conserved, which is also seen in land plants where it is presumed to be at least partly due to RNA-mediated gene conversion (Sloan et al. 2010).

The C-to-U editing type identified here in *A. kona* mtDNA is mostly restricted to land plant organelles where it is widespread (Fujii and Small 2011). DYW-type PPR proteins outside of land plants are intriguing. These proteins were recently identified in a scattering of species from across eukaryotes (Knoop and Rüdinger 2010; Iyer et al. 2011; Schallenberg-Rüdinger et al. 2013), mostly in species noted for their high levels of horizontally acquired genes, for example, *Acanthamoeba* (Clarke et al. 2013), *Naegleria* (Fritz-Laylin et al. 2010), *Physarum* (Watkins and Gray 2008), and *Adineta* (Gladyshev et al. 2008). Phylogenetic signals for possible origins of these HGTs are weak as the proteins are not well conserved in sequence. Nonetheless, there is strong support for lineage-specific expansion of these proteins in *A. kona* and *N. gruberi.* These two gene families also do not appear to group together but rather the DYW-type PPR proteins of *Acrasis* and *Naegleria* group strongly with *Physarum* and rotifer, respectively, suggesting that the two Heteroloboseans are either the source or the recipients of multiple independent HGT events. At least three strong candidate sites for C-to-U RNA editing were also predicted among protein-coding sequences in *Tsukubamonas* mtDNA (data not shown), which still needs further experimental proof. Nevertheless, it suggests that ancient HGT of DYW-type PPR proteins within certain discobid lineages might be more frequent than currently observed.

## Concluding Remarks

The *A. kona* mitochondrial genome shows a number of unusual phenomena that may or may not be linked. The genome appears to be extreme in many different aspects: It has lost over 40% of its annotated coding capacity, massively rearranged its genome, become extremely AT rich and acquired ten novel ORFs that constitute over a quarter of the total mtDNA. This is in striking contrast to its sister lineage, *Naegleria* spp., whose mtDNA appears to closely resemble that of the *Acrasis* + *Naegleria* LCA. This considerably narrows the time frame within which these changes occurred. Thus, it should be possible to dissect some of these dynamic processes by examining additional heterolobosean mtDNAs.

## Supplementary Material

Supplementary tables S1–S5 and figures S1–S7 are available at *Genome Biology and Evolution* online (http://www.gbe.oxfordjournals.org/).

Supplementary Data
