# Supplementary material for: Missing Genes, Multiple ORFs, and C-to-U Type RNA Editing in Acrasis kona (Heterolobosea, Excavata) Mitochondrial DNA
Source: Genome Biol Evol. 2014 Aug 21;6(9):2240–57. doi: 10.1093/gbe/evu180 (PMC4202320; doi:10.1093/gbe/evu180)
Supplement: Supplementary Data [file supp_6_9_2240__index.html]

Missing Genes, Multiple ORFs and C-to-U Type RNA Editing in Acrasis kona (Heterolobosea, Excavata) Mitochondrial DNA — Missing Genes, Multiple ORFs, and C-to-U Type RNA Editing in Acrasis kona (Heterolobosea, Excavata) Mitochondrial DNA — Supplementary Data 

# Missing Genes, Multiple ORFs, and C-to-U Type RNA Editing in *Acrasis kona* (Heterolobosea, Excavata) Mitochondrial DNA

## Supplementary Data

files

**Files in this Data Supplement:**

- Supplementary Data - pdf file
