# Supplementary material for: Missing Genes, Multiple ORFs, and C-to-U Type RNA Editing in Acrasis kona (Heterolobosea, Excavata) Mitochondrial DNA
Source: Genome Biol Evol. 2014 Aug 21;6(9):2240–57. doi: 10.1093/gbe/evu180 (PMC4202320; doi:10.1093/gbe/evu180)
Supplement: Supplementary Data [file supp_evu180_Supp_GBE_No.140527_revise_v2.pdf]

Supplementary Information

Missing Genes, Multiple ORFs and C-to-U Type RNA Editing in *Acrasis kona* (Heterolobosea, Excavata) Mitochondrial DNA

Cheng-Jie Fu[*,1], Sanea Sheikh[1], Wei Miao[2], Siv G. E. Andersson[3], Sandra L. Baldauf[*,1]

[1] Program in Systematic Biology, Department of Organismal Biology, Evolutionary Biology Centre, Uppsala University, Uppsala 75236, Sweden

[2] Key Laboratory of Aquatic Biodiversity and Conservation, Institute of Hydrobiology, Chinese Academy of Sciences, Wuhan 430072, China

[3] Department of Molecular Evolution, Cell and Molecular Biology, Science for Life Laboratory, Biomedical Centre, Uppsala University, Uppsala 75105, Sweden

Contents

A. Supplementary Tables **3**

B. Supplementary Figures **9**

C. References **19**

Supplementary Tables List

Table S1. Primers used in this study.

Table S2. Pair-wise sequence identity of mitochondrial protein-coding genes (OXPHOS pathway) in representative discobids.

Table S3. GC content of mtDNA encoded and 11 putative nuclear encoded mitochondrial protein (NcMt) genes in *Acrasis kona.*

Table S4. Posterior predictive tests of compositional heterogeneity in concatenated alignments of 19 mitochondrial proteins.

Table S5. Comparison of 11 putative nuclear DNA encoded mitochondrial proteins (NcMt) in *Acrasis kona* and their counterparts in the *Naegleria* spp. mtDNAs.

Supplementary Figures List

Figure S1. A schematic map of gap-closing of *Acrasis kona* mtDNA based on long-rang PCR.

Figure S2. Tandemly repeated regions in *Acrasis kona* mtDNA.

Figure S3. Comparison of ribosomal protein operon gene order among representative α-proteobacteria and jakobids.

Figure S4. A split *rps3* gene in *Acrasis kona* mtDNA.

Figure S5. Assembly coverage plots for the 11 putative nuclear encoded mitochondrial protein (NcMt) genes in the *Acrasis kona* nuclear contigs.

Figure S6. Phylogenies of 11 putative *Acrasis kona* nuclear encoded mitochondrial proteins.

Figure S7. Bayesian phylogeny of the E/E+/DYW domain of the DYW-type PPR proteins.

Table S1. Primers used in this study.

Name	Primer (5'-3')
**GAP_CLOSE(1-4)**	
GAP1_F1	TGGGTGCAGCATTTTTAGGACA
GAP1_R1	TCACATTCACCAGCTTGATCACAT
GAP1_nested_F2	CATCAATGGTATTGGGGTTATGG
GAP1_nested_R2	TTGTAGCACATGCAATTACTGGTTT
GAP2_F1	TGTGATCAAGCTGGTGAATGTGATTT
GAP2_R1	GCACCTGCACCTTTGGTTTTCC
GAP2_nested_F2	TGATCAAGCTGGTGAATGTGA
GAP2_nested_R2	TCCTCCATGCCACTTCCACTTT
GAP3_F1	GGAAAACCAAAGGTGCAGGTGCT
GAP3_R1	TGCCCCTGTTGTATCTGTTGCTC
GAP3_nested_F2	TGATAGTCAAGCAGAAAGAGCA
GAP3_nested_R2	TCTAATATGTTGTTTAATTTTTCACT
GAP4_F1	GCAACAGATACAACAGGGGCAAGA
GAP4_R1	CGAACTCCCGACTTTTGGAGTGA
GAP4_nested_F2	AGAGTGTTAGGTCCAGTATGTACAGA
GAP4_nested_R2	TGCTTTCTATTATCTTCCAATTTACCT
**RT-PCR**	
nad1_est_F1	TCTGTTCAAAGAAGAAGAGGACCA
nad1_est_R1	AGCAAAACCCATTCCACTAAA
nad1_est_F2	GCCGATAGCTGATGGTGTAAAA
nad1_est_R2	AAAGCAAAACCCATTCCACT
atp6_est_F1	TTATTATTTGCACAAGGAG
atp6_est_R1	TTTGAGAAAATAGTAGATAAA
atp6_est_F2	ATTGTTTGCTAATTTAGTGG
atp6_est_R2	ATCTTTTGAGAAAATAGTAG
cob_est_F1	TATTATGGTCAAGTGGTGTT
cob_est_R1	TCTGTATGTGATAAATATTCTGG
cob_est_F2	TGCATAGTAATGGTGCAAGT
cob_est_R2	TATTATAACCACCCCATACCCAA
cox3_est_F1	AGAGAGTGTTAAAGGTTGGTTT
cox3_est_R1	TGAAATCCATGAAATCCTGTT
cox3_est_F2	TGTTAGTAGCTTTAATAGAGAGTG
cox3_est_R2	ACAACCTAATCCCATTATCACAGA

Table S2. Pair-wise sequence identity of mitochondrial protein-coding genes (OXPHOS pathway) in representative discobids.

Gene	Sequence Identity (%)			
	Ak/Nf[1]	Ng/Nf	Ag/Jl	Ra/Ha
atp1	49.6	93.8	73.8	86.9
atp3	28.8	78.8	51.1	76.2
atp6	45.05	80.4	64.2	87.1
atp8	21.3	77.3	28.9	74.4
atp9	64.6	98.6	78.1	98.7
cox1	59.05	93	75	89.3
cox2	25.4	86.5	66.3	68.9
cox3	30.4	86.5	66	85
cob	44.65	74	70.7	80.8
nad1	43.9	85.3	70.4	90.1
nad2	30.2	59.6	47.8	80.1
nad3	38.8	86.7	68.1	92.4
nad4	51.15	74.7	68.9	83
nad4L	43.75	90.8	75.5	93.1
nad5	45.25	75.7	63.6	87.8
nad7	/	91.1	75.7	90.5
nad6	30.1	75.1	50.5	74.6
nad8	54.4	91.2	78.4	90.7
nad9	/	74.2	46.3	74.5
nad11	31.25	62.9	52.8	72.3

[1]: Ak: *Acrasis kona*; Ng: *Naegleria gruberi*; Nf: *Naegleria fowleri*; Ag: *Andalucia godoyi*; Ha: *Histiona aroides*; Jl: *Jakoba libera*; Ra: *Reclinomonas americana*.

Table S3. GC content of mtDNA encoded and 11 putative nuclear encoded mitochondrial protein (NcMt) genes in *Acrasis kona.*

mtDNA encoded proteins	Length (aa)	GC	GC1	GC2	GC3	ncDNA encoded proteins	Length (aa)	GC	GC1	GC2	GC3
ORF149	149	0.116	0.134	0.141	0.074	*RPS10*	227	0.398	0.445	0.33	0.419
ORF603	603	0.102	0.109	0.134	0.061	*RPL11*	200	0.395	0.485	0.400	0.300
ORF246	246	0.081	0.061	0.114	0.069	*RPL2*	256	0.422	0.488	0.461	0.316
ORF802	802	0.086	0.105	0.103	0.051	*RPS19*	99	0.377	0.354	0.364	0.414
ORF1592	1592	0.186	0.205	0.190	0.163	*RPL16*	194	0.400	0.412	0.371	0.418
ORF376	376	0.188	0.277	0.215	0.072	*RPS12*	172	0.403	0.430	0.448	0.331
ORF61	61	0.109	0.148	0.098	0.082	*RPS2*	254	0.419	0.425	0.331	0.500
ORF399	399	0.183	0.278	0.190	0.080	*NAD7*	432	0.464	0.475	0.373	0.544
ORF253	253	0.158	0.292	0.123	0.059	*NAD9*	195	0.400	0.451	0.297	0.451
ORF69	69	0.121	0.188	0.130	0.043	*SDH2*	318	0.437	0.406	0.374	0.531
NAD1	380	0.214	0.268	0.289	0.084	*COX11*	283	0.395	0.445	0.350	0.389
NAD2	518	0.133	0.166	0.195	0.039	Average	/	0.417	0.445	0.370	0.436
NAD3	124	0.161	0.218	0.202	0.065						
NAD4	465	0.164	0.196	0.237	0.060						
NAD4L	88	0.155	0.159	0.227	0.080						
NAD5	637	0.152	0.196	0.209	0.052						
NAD6	230	0.161	0.230	0.187	0.065						
NAD8	143	0.233	0.336	0.322	0.042						
NAD11	591	0.148	0.198	0.193	0.054						
COB	478	0.196	0.251	0.282	0.054						
COX1	663	0.248	0.324	0.326	0.095						
COX2	307	0.228	0.332	0.290	0.062						
COX3	316	0.190	0.282	0.222	0.066						
ATP1	557	0.266	0.357	0.343	0.097						
ATP3	333	0.172	0.228	0.207	0.081						
ATP6	261	0.143	0.188	0.199	0.042						
ATP8	126	0.124	0.127	0.167	0.079						
ATP9	72	0.296	0.361	0.431	0.097						
RPS3_a	966	0.124	0.130	0.115	0.125						
RPS3_b	1133	0.092	0.117	0.102	0.058						
RPS7	197	0.142	0.157	0.157	0.112						
RPS8	124	0.124	0.169	0.153	0.048						
RPS13	136	0.137	0.169	0.162	0.081						
RPS14	98	0.201	0.194	0.276	0.133						
RPL5	274	0.145	0.208	0.172	0.055						
RPL6	226	0.165	0.199	0.168	0.128						
RPL14	121	0.240	0.289	0.339	0.091						
Average	/	0.160	0.203	0.195	0.082						

Table S4. Posterior predictive tests of compositional heterogeneity in concatenated alignments of 19 mitochondrial proteins.

Taxa	Original dataset			Recoding using 6 Dayhoff groups			GenBank accession number
	z-score	p-value	Significance	z-score	p-value	Significance	
Acrasis kona	19.041	0	*	21.938	0	*	KJ679272
Naegleria gruberi	13.514	0	*	18.449	0	*	AF288092
Acanthamoeba castellanii	16.058	0	*	19.052	0	*	NC_001637
Agrobacterium radiobacter	8.507	0.004	*	4.673	0.01	*	NC_011985, NC_011983
Allomyces macrogynus	6.614	0	*	3.017	0.006	*	NC_001715
Andalucia godoyi	5.544	0.006	*	-1.126	0.896		KC353352
Azospirillum brasilense	8.385	0	*	4.941	0.004	*	NC_016617
Bigelowiella natans	7.994	0.008	*	6.811	0.004	*	HQ840955
Bradyrhizobium japonicum	7.958	0.008	*	6.435	0.004	*	NC_004463
Capsaspora owczarzaki	8.574	0	*	5.958	0.008	*	KC573038
Chaetosphaeridium globosum	2.876	0.024	*	1.479	0.084		NC_004118
Chattonella marina	10.989	0	*	0.896	0.158		NC_013837
Chondrus crispus	9.947	0	*	3.13	0.006	*	NC_001677
Cyanidioschyzon merolae	13.561	0	*	7.577	0	*	NC_000887
Cyanophora paradoxa	15.842	0	*	6.322	0.004	*	NC_017836
Desmarestia viridis	5.165	0.008	*	1.21	0.116		NC_007684
Dictyostelium discoideum	15.055	0	*	19.753	0	*	NC_000895
Ehrlichia canis	11.884	0	*	5.829	0.002	*	NC_007354
Emiliania huxleyi	6.772	0	*	1.073	0.132		NC_005332
Geodia neptuni	3.431	0	*	3.624	0.002	*	NC_006990
Glaucocystis nostochinearum	12.845	0	*	9.692	0	*	NC015117
Hartmannella vermiformis	10.794	0.002	*	11.958	0	*	NC_013986
Hemiselmis andersenii	4.129	0.004	*	0.124	0.368		NC_010637
Histiona aroides	3.225	0.006	*	0.08	0.432		KC353353
Jakoba bahamiensis	2.603	0.026	*	-0.994	0.842		KC353354
Jakoba libera	6.96	0.006	*	0.753	0.216		KC353355
Magnetospirillum magnetotacticum	7.81	0.006	*	7.676	0.004	*	NZ_AAAP00000000
Magnetococcus sp.	8.127	0.008	*	4.886	0.01	*	NC_008576
Malawimonas californiana	14.887	0	*	9.162	0	*	unpublished
Malawimonas jakobiformis	15.177	0	*	5.252	0	*	NC_002553
Marchantia polymorpha	1.434	0.082		0.887	0.174		NC_001660
Mezorhizobium loti	6.306	0.008	*	5.896	0.008	*	NC_002682, NC_002679, NC_002678
Mesostigma viride	9.064	0.002	*	1.211	0.116		NC_008240
Micavibrio aeruginosavorus	9.456	0.008	*	3.445	0.018	*	NC_016026

Midichloria mitochondrii	4.18	0.008	*	0.146	0.34		NC_015722
Monosiga brevicollis	5.684	0.002	*	2.249	0.034	*	NC_004309
Neorickettsia sennetsu	4.089	0.004	*	3.545	0.008	*	NC_007798
Nephroselmis olivacea	1.882	0.042	*	1.205	0.11		NC_008239
Novosphingobium aromaticivorans	6.526	0.016	*	9.517	0.004	*	NC_007794
Orientia tsutsugamushi str. Ideda	17.564	0	*	8.216	0	*	NC_010793
Pavlova lutheri	6.215	0.002	*	-0.773	0.784		HQ908424
Phytophthora infestans	14.909	0	*	14.167	0	*	NC_002387
Porphyra purpurea	4.654	0	*	-0.552	0.716		NC_002007
Prototheca wickerhamii	1.884	0.04	*	0.96	0.168		NC_001613
Reclinomonas americana	1.288	0.106		-0.473	0.652		NC_001823
Rhizopus oryzae	3.762	0.014	*	4.7	0.008	*	NC_006836
Rhodospirillum rubrum	10.257	0.002	*	8.326	0	*	NC_007643
Rhodospirillum centenum	9.187	0.004	*	5.575	0.006	*	NC_011420
Rhodomonas salina	13.292	0	*	20.171	0	*	NC_002572
Rickettsia prowazekii str. Madrid	13.246	0	*	6.81	0	*	NC_000963
Seculamonas ecuadoriensis	0.507	0.258		0.053	0.432		KC353359
Thalassiosira pseudonana	9.779	0	*	2.922	0.014	*	NC_007405
Tistrella mobilis	8.045	0.004	*	6.099	0.008	*	NC_017966
Alphaproteobacterium BAL199	6.301	0.006	*	5.105	0.004	*	NZ_ABHC01000000
Global test	33.573	0		22.297	0		

Table S5. Comparison of 11 putative nuclear encoded mitochondrial proteins (NcMt) in *Acrasis kona* and their counterparts in the *Naegleria* spp. mtDNAs.

Protein	Species	Length (aa)	TargetP 1.1		MitoProt II		Predotar	Significance[1]
			P value	Cleavage length(aa)	P value	Cleavage length(aa)	P value	
RPS10	*A. kona*	227	0.647	17	0.900	13	0.76	+++
	N. gruberi	220	0.381	26	0.999	77	0.26	+
	N. fowleri	77	0.393	n.a.	0.150	n.a.	0.65	+
RPL11	*A. kona*	200	0.669	16	0.978	54	0.60	+++
	N. gruberi	148	0.272	n.a.	0.181	n.a.	0.32	−
	N. fowleri	145	0.390	82	0.625	n.a.	0.22	+
RPL2	*A. kona*	256	0.913	62	0.994	52	0.77	+++
	N. gruberi	268	0.310	n.a.	0.565	n.a.	0.10	+
	N. fowleri	266	0.182	n.a.	0.930	n.a.	0.35	+
RPS19	*A. kona*	99	0.708	14	0.981	n.a.	0.56	+++
	N. gruberi	88	0.633	54	0.879	n.a.	0.05	++
	N. fowleri	87	0.387	n.a.	0.675	n.a.	0.02	+
RPL16	*A. kona*	194	0.681	110	0.992	45	0.83	+++
	N. gruberi	141	0.412	n.a.	0.939	n.a.	0.47	+
	N. fowleri	141	0.648	16	0.989	n.a.	0.47	++
RPS12	*A. kona*	172	0.919	49	0.999	160	0.86	+++
	N. gruberi	128	0.294	n.a.	0.932	97	0.33	+
	N. fowleri	128	0.342	n.a.	0.364	15	0.47	−
RPS2	*A. kona*	254	0.341	n.a.	0.849	101	0.0	+
	N. gruberi	264	0.206	n.a.	0.950	n.a.	0.16	+
	N. fowleri	262	0.192	n.a.	0.566	27	0.03	+
NAD7	*A. kona*	432	0.674	30	0.586	n.a.	0.83	+++
	N. gruberi	395	0.094	n.a.	0.082	38	0.0	−
	N. fowleri	395	0.066	n.a.	0.155	n.a.	0.0	−
NAD9	*A. kona*	195	0.334	n.a.	0.817	n.a.	0.50	++
	N. gruberi	186	0.551	36	0.911	n.a.	0.01	++
	N. fowleri	185	0.294	23	0.636	23	0.0	+
SDH2	*A. kona*	318	0.838	26	0.933	40	0.65	+++
	N. gruberi	270	0.133	n.a.	0.113	n.a.	0.02	−
	N. fowleri	270	0.138	22	0.756	n.a.	0.04	+
COX11	*A. kona*	283	0.804	14	0.948	22	0.86	+++
	N. gruberi	222	0.015	21	0.327	n.a.	0.01	−
	N. fowleri	224	0.008	25	0.076	n.a.	0.01	−

[1]: (+): one of the predictions have P>0.50; (−): none of the predictions have P>0.50

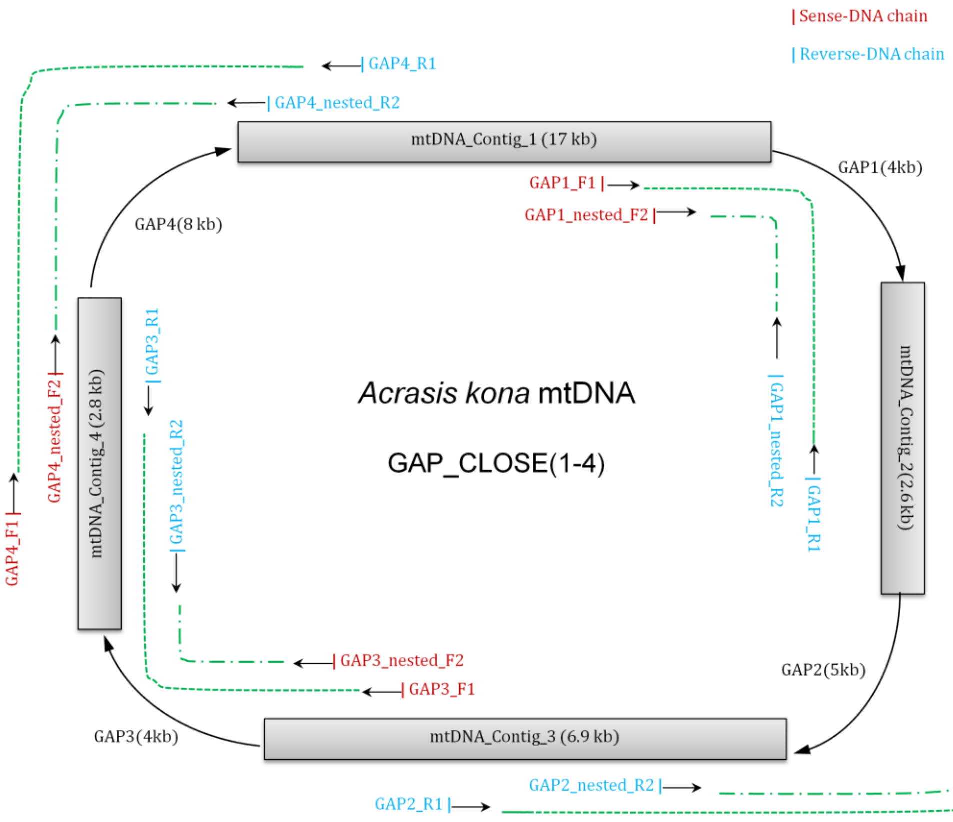

Fig S1. A schematic map of gap-closing of *Acrasis kona* mtDNA based on long-rang PCR.

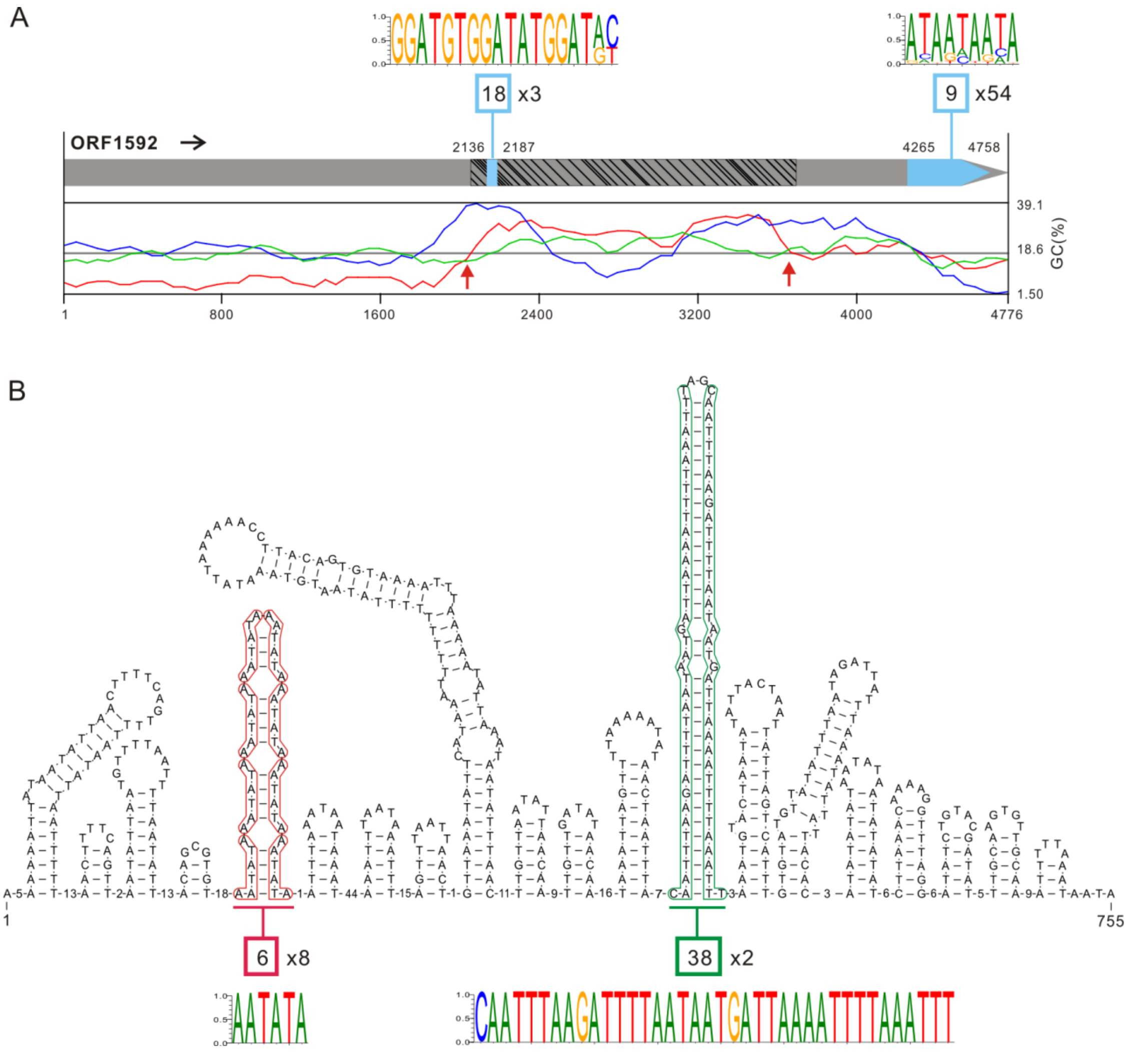

Fig S2. Tandemly repeated regions in *Acrasis kona* mtDNA. (A) Two tandem repeats occur in the largest putative protein coding gene (ORF1592). The region of heterogeneous GC content is shaded. GC Frame plot of GC content at the 1st, 2^{ed} and 3rd codon positions is shown below with a window size of 400. (B) The nucleotide repeat and the potential secondary structures in the largest noncoding region (755 bp) of *A. kona* mtDNA. The period size of nucleotide repeat is indicated in the box.

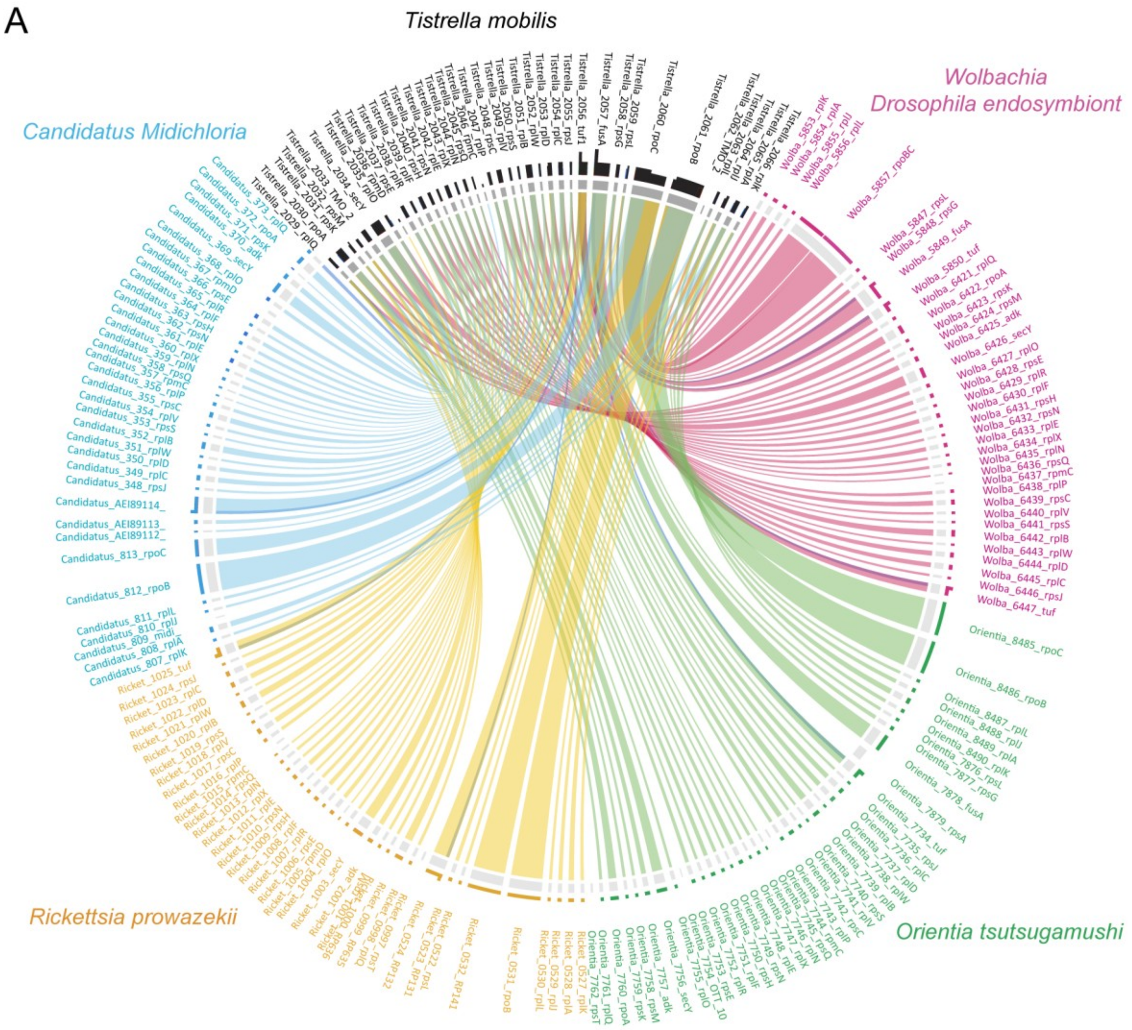
A
Tistrella mobilis
Wolbachia
Drosophila endosymbiont
Candidatus Midichloria
Rickettsia prowazekii
Orientia tsutsugamushi

(Fig S3)

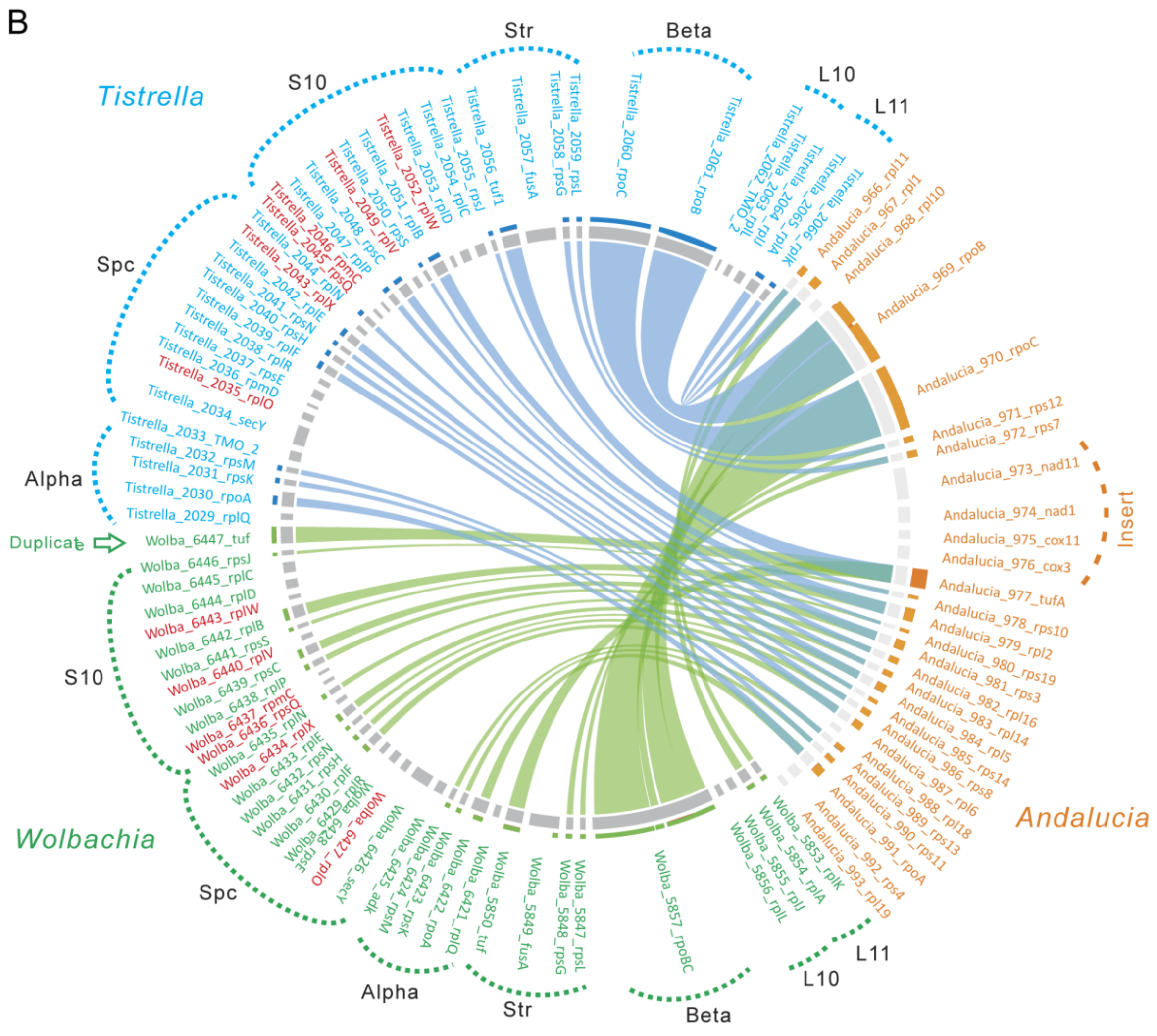

(Fig S3. Continued)

Fig S3. Comparison of ribosomal protein operon gene order among representative α-proteobacteria and jakobids. (A) Sequences identified by BLASTp search of four *Rickettsiales* species against the free-living α-proteobacteria *Tistrella mobilis* (NC_017956) as the reference with a threshold of 1e^{-10}. (B) Sequences identified by BLASTp search of *Rickettsiales* species *Wolbachia* and *Tistrella mobilis* against the jakobid species *Andalucia goyodi* (KC353352) as the reference with a threshold of 1e^{-10}. The range of each protein operon is indicated with dotted line. Names of genes with discrepancy of their presence in the *Rickettsiales* species in comparision with Burger et al. 2013 are indicated in red. Genbank accession numbers of the genomes: NC_002978 (*Wolbachia* endosymbiont of *Drosophila melanogaster*), NC_010793 (*Orientia tsutsugamushi*), NC_000963 (*Rickettsia prowazekii*) and NC_015722 (*Candidatus Midichloria mitochondrii*).

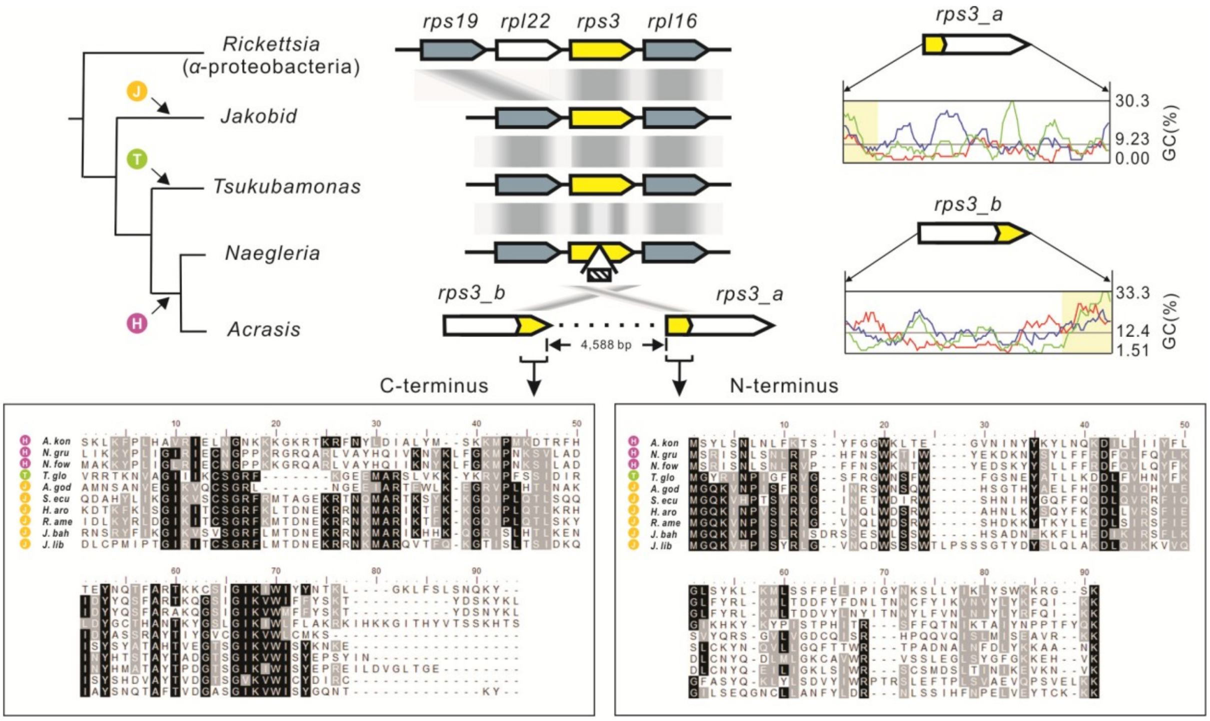

Fig S4. A split *rps3* gene in *Acrasis kona* mtDNA. The *rps3* gene organization in the α-proteobacteria (*Rickettsia*), Jakobida, Tsukubamonadida and Heterolobosea are shown next to a schematic species phylogeny. The *Naegleria rps3* gene has long insertions in the middle that are poorly aligned. The *Acrasis rps3* gene is split into two ORFs (*rps3_a* and *rps3_b*), both with terminal extensions. Grey intensity reflects the degree of similarity between the gene orthologs. Multiple sequence alignments for the regions corresponding to the two halves of *rps3* are shown below in boxes. Residues with 100% identity are shaded in black, with over 60% similarity shaded in grey. The GC content of *Acrasis rps3_a* and *rps3_b* at each codon position is shown as determined by GC Frame plot (window size 200).

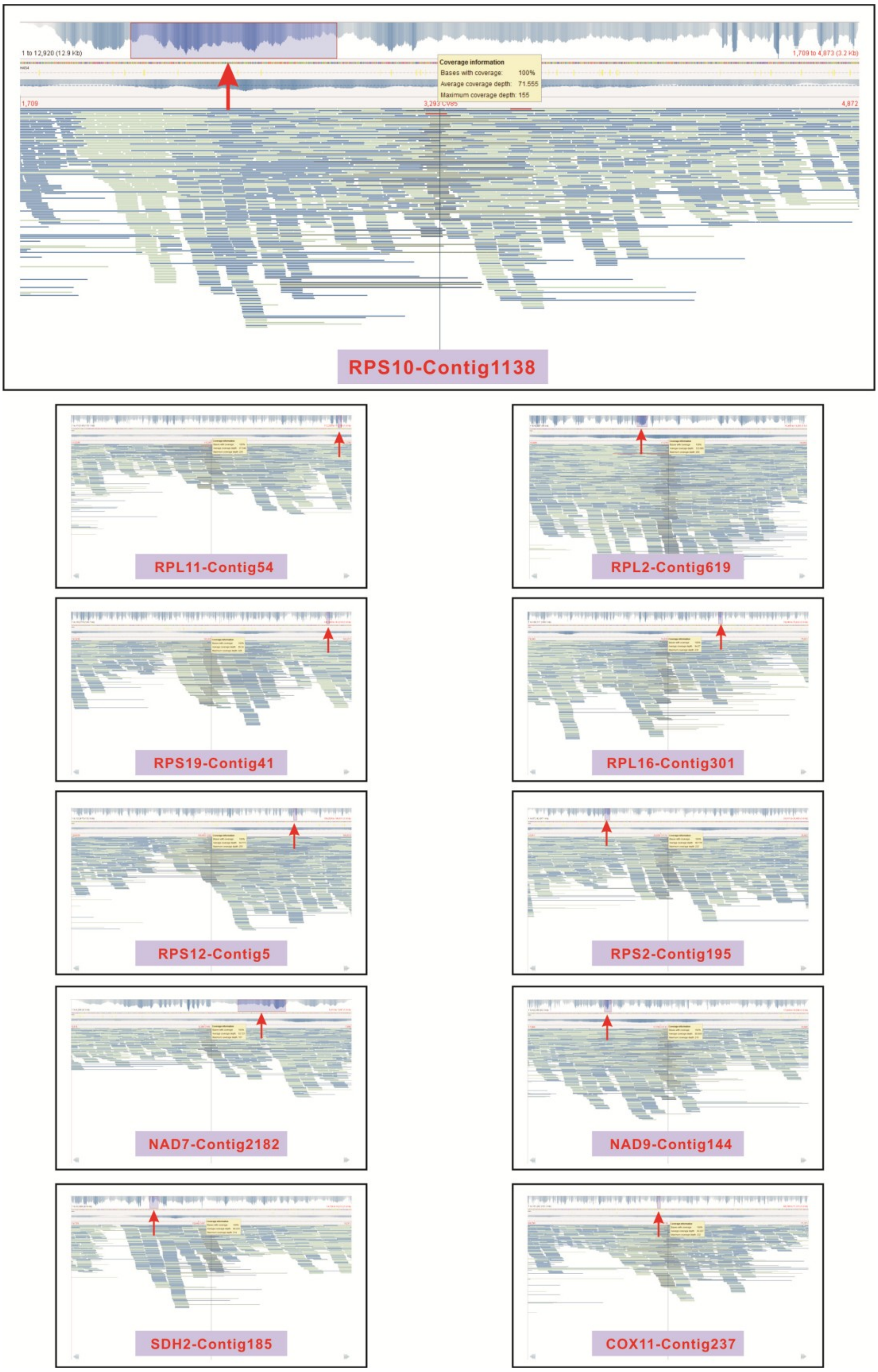

Fig S5. Assembly coverage plots for the 11 putative nuclear encoded mitochondrial protein (NcMt) genes in the *Acrasis kona* nuclear contigs. The corresponding loci of each NcMt gene candidate is indicated with red arrow.

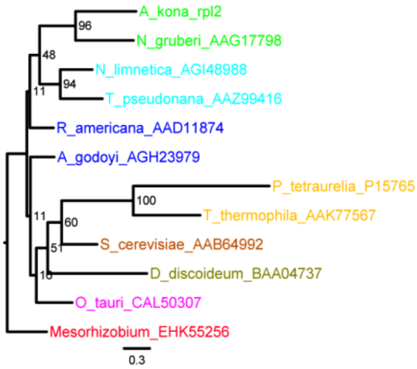

A. rpl12 (223 aligned positions)

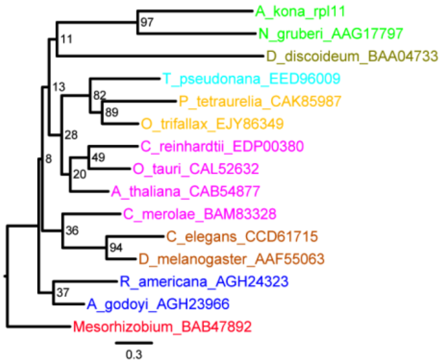

B. rpl11 (126 aligned positions)

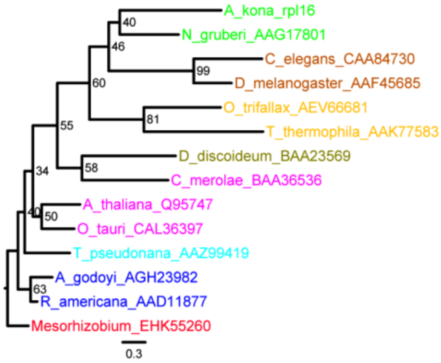

C. rpl16 (118 aligned positions)

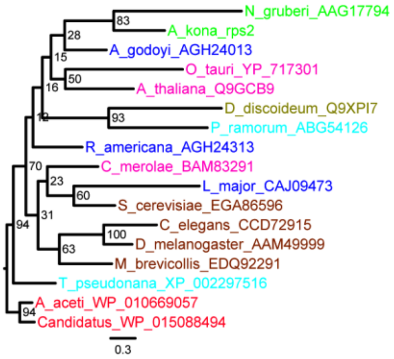

D. rps2 (171 aligned positions)

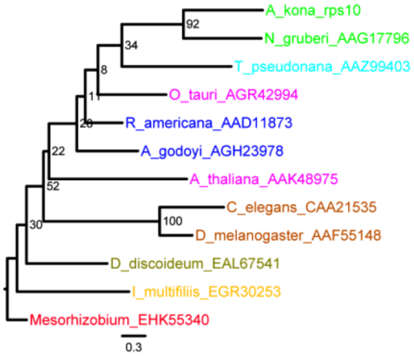

E. rps10 (99 aligned positions)

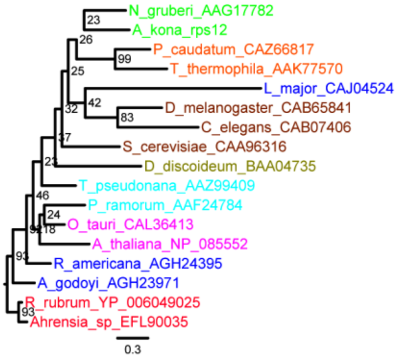

F. rps12 (121 aligned positions)

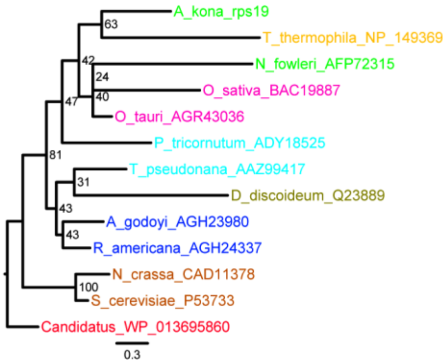

G. rps19 (80 aligned positions)

(Fig S6.)

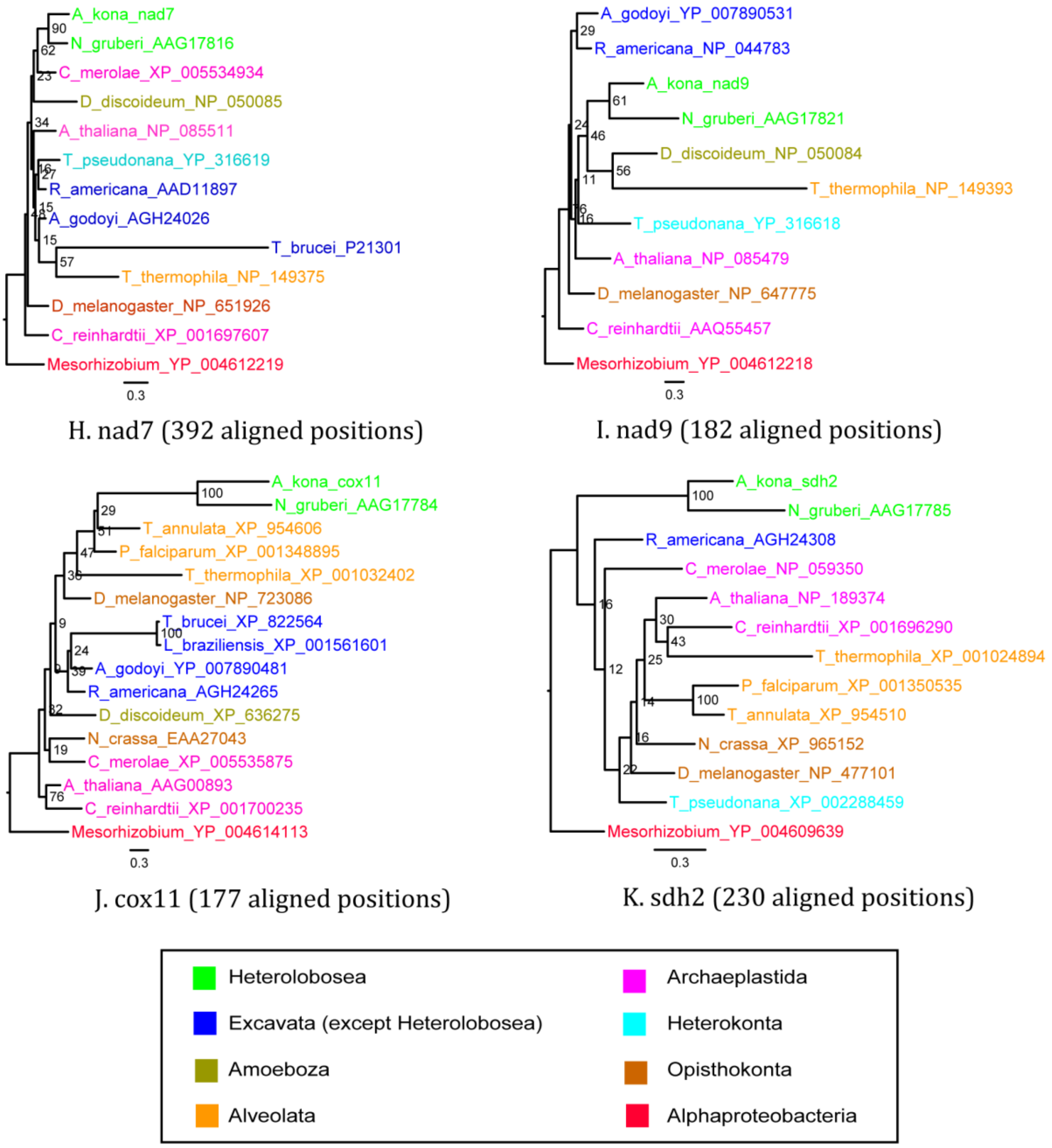

(Fig S6. Continued)

Fig S6. Phylogenies of 11 *Acrasis kona* putative nuclear-encoded mitochondrial proteins (NcMt). Trees were derived by maximum likelihood analysis of amino acid sequences with the number of aligned positions for individual data sets indicated below the respective trees. Numbers indicate bootstrap values from 1000 replicates of rapid maximum likelihood bootstrapping with RAxML v7.3.3 (Stamatakis 2006). Taxa were selected to give a broad sampling of eukaryotes based on (Desmond et al. 2011) but with emphasis on Discoba. Taxon labels are color coded as indicate at the bottom right. Trees are drawn to scale as indicated by the scale bars.

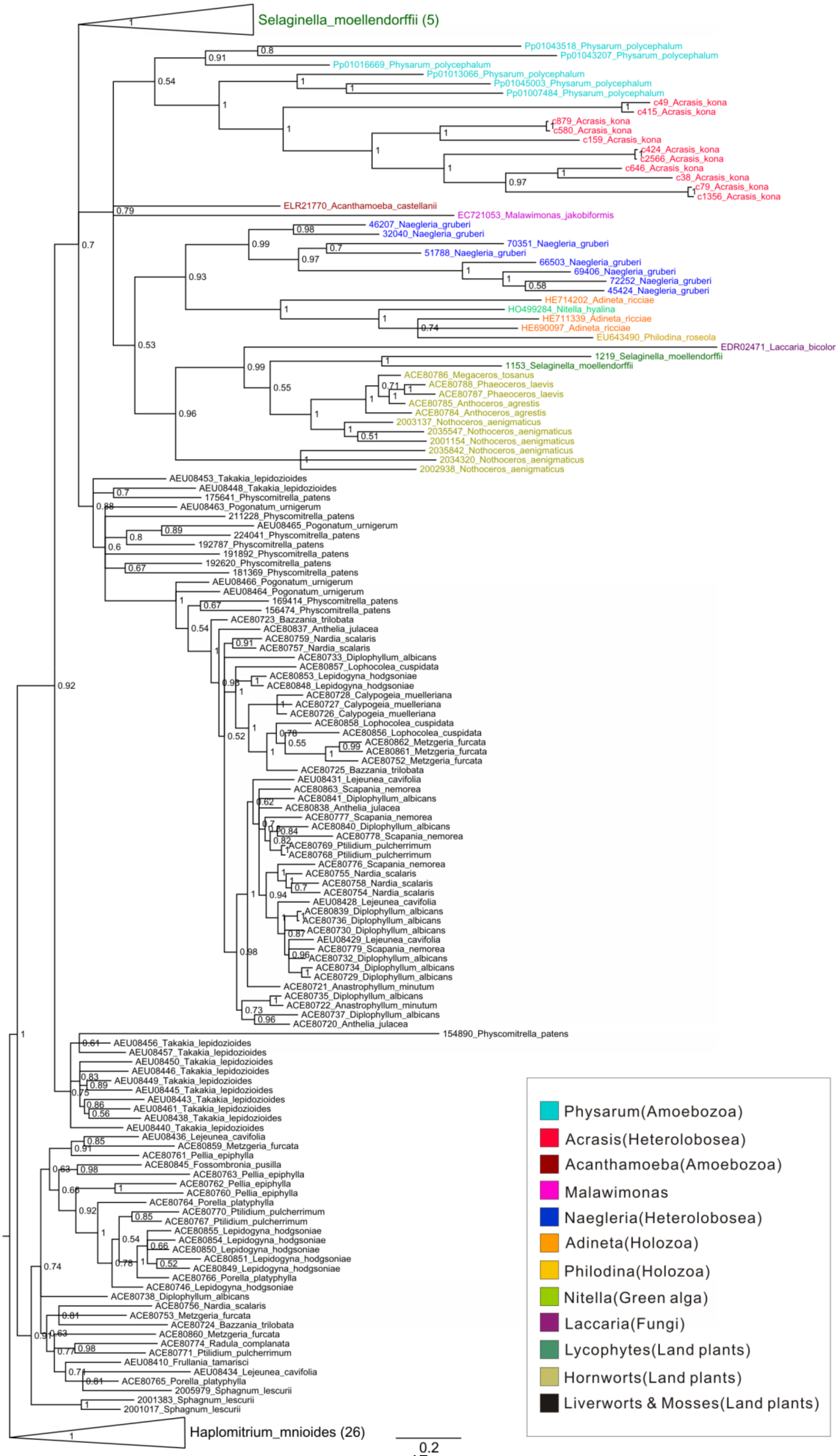

Selaginella_moellendorffii (5)
Pp01043518_Physarum_polycephalum
Pp01043207_Physarum_polycephalum
Pp01016669_Physarum_polycephalum
Pp01013066_Physarum_polycephalum
Pp01045003_Physarum_polycephalum
Pp01007484_Physarum_polycephalum
c49_Acrasis_kona
c415_Acrasis_kona
c879_Acrasis_kona
c580_Acrasis_kona
c159_Acrasis_kona
c424_Acrasis_kona
c2566_Acrasis_kona
c646_Acrasis_kona
c38_Acrasis_kona
c79_Acrasis_kona
c1356_Acrasis_kona
ELR21770_Acanthamoeba_castellanii
EC721053_Malawimonas_jakobiformis
46207_Naegleria_gruberi
32040_Naegleria_gruberi
70351_Naegleria_gruberi
51788_Naegleria_gruberi
66503_Naegleria_gruberi
69406_Naegleria_gruberi
72252_Naegleria_gruberi
45424_Naegleria_gruberi
HE714202_Adineta_ricciae
HO499284_Nitella_hyalina
HE711339_Adineta_ricciae
HE690097_Adineta_ricciae
EU643490_Philodina_roseola
EDR02471_Laccaria_bicolor
1219_Selaginella_moellendorffii
1153_Selaginella_moellendorffii
ACE80786_Megaceros_tosanus
ACE80788_Phaeoceros_laevis
ACE80787_Phaeoceros_laevis
ACE80785_Anthoceros_agrestis
ACE80784_Anthoceros_agrestis
2003137_Nothoceros_aenigmaticus
2035547_Nothoceros_aenigmaticus
2001154_Nothoceros_aenigmaticus
2035842_Nothoceros_aenigmaticus
2034320_Nothoceros_aenigmaticus
2002938_Nothoceros_aenigmaticus
AEU08453_Takakia_lepidozioides
AEU08448_Takakia_lepidozioides
175641_Physcomitrella_patens
AEU08463_Pogonatum_urnigerum
211228_Physcomitrella_patens
AEU08465_Pogonatum_urnigerum
224041_Physcomitrella_patens
192787_Physcomitrella_patens
191892_Physcomitrella_patens
192620_Physcomitrella_patens
181369_Physcomitrella_patens
AEU08466_Pogonatum_urnigerum
AEU08464_Pogonatum_urnigerum
169414_Physcomitrella_patens
156474_Physcomitrella_patens
ACE80723_Bazzania_trilobata
ACE80837_Anthelia_julacea
ACE80759_Nardia_scalaris
ACE80757_Nardia_scalaris
ACE80733_Diplophyllum_albicans
ACE80857_Lophocolea_cuspidata
ACE80853_Lepidogyna_hodgsoniae
ACE80848_Lepidogyna_hodgsoniae
ACE80728_Calypogeia_muelleriana
ACE80727_Calypogeia_muelleriana
ACE80726_Calypogeia_muelleriana
ACE80858_Lophocolea_cuspidata
ACE80856_Lophocolea_cuspidata
ACE80862_Metzgeria_furcata
ACE80861_Metzgeria_furcata
ACE80752_Metzgeria_furcata
ACE80725_Bazzania_trilobata
AEU08431_Lejeunea_cavifolia
ACE80863_Scapania_nemorea
ACE80841_Diplophyllum_albicans
ACE80838_Anthelia_julacea
ACE80777_Scapania_nemorea
ACE80840_Diplophyllum_albicans
ACE80778_Scapania_nemorea
ACE80769_Ptilidium_pulcherrimum
ACE80768_Ptilidium_pulcherrimum
ACE80776_Scapania_nemorea
ACE80755_Nardia_scalaris
ACE80758_Nardia_scalaris
ACE80754_Nardia_scalaris
AEU08428_Lejeunea_cavifolia
ACE80839_Diplophyllum_albicans
ACE80736_Diplophyllum_albicans
ACE80730_Diplophyllum_albicans
AEU08429_Lejeunea_cavifolia
ACE80779_Scapania_nemorea
ACE80732_Diplophyllum_albicans
ACE80734_Diplophyllum_albicans
ACE80729_Diplophyllum_albicans
ACE80721_Anastrophyllum_minutum
ACE80735_Diplophyllum_albicans
ACE80722_Anastrophyllum_minutum
ACE80737_Diplophyllum_albicans
ACE80720_Anthelia_julacea
154890_Physcomitrella_patens
AEU08456_Takakia_lepidozioides
AEU08457_Takakia_lepidozioides
AEU08450_Takakia_lepidozioides
AEU08446_Takakia_lepidozioides
AEU08449_Takakia_lepidozioides
AEU08445_Takakia_lepidozioides
AEU08443_Takakia_lepidozioides
AEU08461_Takakia_lepidozioides
AEU08438_Takakia_lepidozioides
AEU08440_Takakia_lepidozioides
AEU08436_Lejeunea_cavifolia
ACE80859_Metzgeria_furcata
ACE80761_Pellia_epiphylla
ACE80845_Fossombronia_pusilla
ACE80763_Pellia_epiphylla
ACE80762_Pellia_epiphylla
ACE80760_Pellia_epiphylla
ACE80764_Porella_platyphylla
ACE80770_Ptilidium_pulcherrimum
ACE80767_Ptilidium_pulcherrimum
ACE80855_Lepidogyna_hodgsoniae
ACE80854_Lepidogyna_hodgsoniae
ACE80850_Lepidogyna_hodgsoniae
ACE80851_Lepidogyna_hodgsoniae
ACE80849_Lepidogyna_hodgsoniae
ACE80766_Porella_platyphylla
ACE80746_Lepidogyna_hodgsoniae
ACE80738_Diplophyllum_albicans
ACE80756_Nardia_scalaris
ACE80753_Metzgeria_furcata
ACE80724_Bazzania_trilobata
ACE80860_Metzgeria_furcata
ACE80774_Radula_complanata
ACE80771_Ptilidium_pulcherrimum
AEU08410_Frullania_tamarisci
AEU08434_Lejeunea_cavifolia
ACE80765_Porella_platyphylla
2005979_Sphagnum_lescurii
2001383_Sphagnum_lescurii
2001017_Sphagnum_lescurii
Haplomitrium_mnioides (26)
0.2
Physarum(Amoebozoa)
Acrasis(Heterolobosea)
Acanthamoeba(Amoebozoa)
Malawimonas
Naegleria(Heterolobosea)
Adineta(Holozoa)
Philodina(Holozoa)
Nitella(Green alga)
Laccaria(Fungi)
Lycophytes(Land plants)
Hornworts(Land plants)
Liverworts & Mosses(Land plants)

Fig S7. Bayesian phylogeny of the E/E+/DYW domain of the DYW-type PPR proteins. Taxa include a broad sampling of early branching land plants [Liverworts (Haplomitriopsida and Jungermanniopsida), Mosses (Bryopsida, Polytrichopsida, Sphagnopsida and Takakiopsida) , Hornworts (Anthocerotopsida) and Lycophytes (Isoetopsida)], and those outside land plants [Heterolobosea (*Acrasis kona*; *Naegleria gruberi*), Amoebozoa (*Acanthamoeba castellanii*; *Physarum polycephalum*), Metazoa (*Adineta ricciae*; *Philodina roseola*), Fungi (*Laccaria bicolor*), Charophyta (*Nitella hyaline*), *Malawimonas jakobiformis*]. The candidate protein sequences from species outside of land plants were retrieved based on (Iyer et al. 2011; Schallenberg-Rudinger et al. 2013). The tree shown was derived using the program PhyloBayes v1.4 (Lartillot et al, 2009) using an alignment of 201 universally-aligned amino acid positions. Values correspond to posterior probabilities; all nodes with posterior probability less than 0.5 are collapsed. Taxon labels include Genbank accession numbers and are color coded according to the key at the bottom right. The tree is drawn to scale as indicated by the scale bar.

References

1. Burger G, Gray MW, Forget L, Lang BF. 2013. Strikingly bacteria-like and gene-rich mitochondrial genomes throughout jakobid protists. Genome Biol Evol 5: 418-438.
2. Desmond E, Brochier-Armanet C, Forterre P, Gribaldo S. 2011. On the last common ancestor and early evolution of eukaryotes: reconstructing the history of mitochondrial ribosomes. Res Microbiol 162: 53-70.
3. Iyer LM, Zhang D, Rogozin IB, Aravind L. 2011. Evolution of the deaminase fold and multiple origins of eukaryotic editing and mutagenic nucleic acid deaminases from bacterial toxin systems. Nucleic Acids Res 39: 9473-9497.
4. Lartillot N, Lepage T, Blanquart S. 2009. PhyloBayes 3: a Bayesian software package for phylogenetic reconstruction and molecular dating. Bioinformatics 25: 2286-2288.
5. Schallenberg-Rudinger M, Lenz H, Polsakiewicz M, Gott JM, Knoop V. 2013. A survey of PPR proteins identifies DYW domains like those of land plant RNA editing factors in diverse eukaryotes. RNA Biol 10: 1549-1556.
6. Stamatakis A. 2006. RAxML-VI-HPC: maximum likelihood-based phylogenetic analyses with thousands of taxa and mixed models. Bioinformatics 22: 2688-2690.
